# Raman signatures of *Cnm*-positive *Streptococcus mutans*: I, the molecular origin of cerebral microbleeds

**DOI:** 10.3389/fmicb.2026.1784125

**Published:** 2026-04-27

**Authors:** Giuseppe Pezzotti, Tetsuya Adachi, Kazunori Kitagawa, Saki Ikegami, Hayata Imamura, Toshiro Yamamoto, Kazu Okuma, Yoshiyuki Matsuo, Wenliang Zhu, Yoshiki Yasukochi, Koichiro Higasa, Kazuhisa Ouhara, Saki Nishihama, Katsuhiro Takeda, Hideki Shiba, Miki Kawada-Matsuo, Hitoshi Komatsuzawa

**Affiliations:** 1 Biomedical Engineering Center, Kansai Medical University, Hirakata, Osaka, Japan; 2 International Center for Biomedical Industrial Promotion, Kansai Medical University, Hirakata, Osaka, Japan; 3 Department of Immunology, Graduate School of Medical Science, Kyoto Prefectural University of Medicine, Kyoto, Japan; 4 Department of Orthopedic Surgery, Tokyo Medical University, Shinjuku-ku, Tokyo, Japan; 5 Department of Molecular Science and Nanosystems, Ca’ Foscari University of Venice, Venice, Italy; 6 Biomarker Disease Laboratory, IRCCS San Camillo Hospital, Venice Lido, Italy; 7 Department of Orthopaedic Surgery, Mie University, Graduate School of Medicine, Tsu, Japan; 8 Department of Dental Medicine, Graduate School of Medical Science, Kyoto Prefectural University of Medicine, Kamigyo-ku, Kyoto, Japan; 9 Department of Microbiology, Kansai Medical University, School of Medicine, Hirakata, Osaka Prefecture, Japan; 10 Ceramic Physics Laboratory, Kyoto Institute of Technology, Sakyo-ku, Matsugasaki, Kyoto, Japan; 11 Department of Genome Analysis, Institute of Biomedical Science, Kansai Medical University, Hirakata, Osaka, Japan; 12 Central Research Center, Institute of Biomedical Science, Kansai Medical University, Hirakata, Osaka Prefecture, Japan; 13 Department of Periodontal Medicine, Graduate School of Biomedical and Health Sciences, Hiroshima University, Minami-ku, Hiroshima, Japan; 14 Department of Biological Endodontics, Graduate School of Biomedical & Health Sciences, Hiroshima University, Minami-ku, Hiroshima, Japan; 15 Department of Bacteriology, Graduate School of Biomedical & Health Sciences, Hiroshima University, Minami-ku, Hiroshima, Japan

**Keywords:** biofilm structure, *Cnm* protein, ischemic stroke, Raman spectroscopy, *Streptococcus mutans*

## Abstract

This study featured a Raman spectroscopic analysis aimed at unfolding structural differences at the molecular scale between *Cnm*-positive and *Cnm*-negative *Streptococcus mutans* bacteria and their respective biofilms. The primary goal of this analysis was to unveil the molecular origin of enhanced virulence and propensity to damage endothelial tissue of strains containing the *Cnm* gene. *In situ* Raman assessments of live biofilm exopolysaccharides, generated upon sucrose addition by *Cnm*-positive and negative cultures, revealed additional features that linked to specific metabolic characteristics. The Raman spectra of *Cnm*-positive and *Cnm*-negative bacterial cells were strikingly different. In the *Cnm*-positive strain, strong signals from a variety of residual oxysulfur molecules, including indoxyl and other sulfates (likely stored in the *Cnm* protein at the bacterial surface), suggested that this strain is capable to exploit sulfur chemistry for anchoring purposes, while simplifying the structure of its peptidoglycan membrane. On the other hand, the *Cnm*-positive strain appeared to have conspicuously lost its capacity of producing and storing glycogen molecules, possibly allocating differently its resources in adaptive advantage for tissue invasive activity. Regarding biofilm structure, the *Cnm*-positive strain produced larger amounts of glucans for a lesser number of cells; however, the ratio between *α*-1,6- and α-1,3-glucan fractions showed no significant variations in comparison to the *Cnm*-negative strain, suggesting that adhesion rather than biofilm strength and impermeability is the main priority in its overall functionality.

## 1 Introduction

The presence of the *Cnm* gene in *Streptococcus mutans* (*S. mutans*) has been linked to increased virulence and to a potential association with systemic conditions such as hemorrhagic stroke (Abranches et al., 2011; Nakano et al., 2011; Naka et al., 2022; Hosoki et al., 2022; Bayraktar et al., 2025; Yamamoto et al., 2025; Matsuoka et al., 2026; Okuda et al., 2025). The *Cnm* gene encodes a collagen-binding protein, which allows *S. mutans* to adhere not just to dental surfaces, but also to other collagen-containing tissues in other parts of the body (Abranches et al., 2011; Nakano et al., 2011; Mieher et al., 2025; Suehiro et al., 2025). This protein has been associated to the bacterium’s ability to adhere to and successively invade endothelial cells, which line blood vessels (Nakano et al., 2011; Aviles-Reyes et al., 2017; Wang, 2011). Recent studies (Abranches et al., 2011; Nakano et al., 2011; Wang et al., 2011; Wardlaw et al., 2013; Tonomura et al., 2016; Aviles-Reyes et al., 2017; Otsugu et al., 2017; Song et al., 2025; Leonov et al., 2024) have demonstrated, both from biochemical and clinical approaches, that individuals with higher prevalence of *Cnm*-positive *S. mutans* (*Cnm*^(+)^*Sm*) are at a statistically greater risk for ischemic stroke, this being a direct consequence of the bacterium’s ability to move from the oral cavity and invade endothelial cells inducing vascular damage (Abranches et al., 2006; Abranches et al., 2011; Xiao and Li, 2025; Fang et al., 2024). While the exact mechanisms for such damage are still being studied, it is hypothesized that *S. mutans* contributes to stroke by first inducing inflammation and damage within blood vessels, and then by creating conditions conducive to clot formation, which can in turn lead to ischemic events, while concurrently triggering immune responses that could further damage vascular tissues (Abranches et al., 2006; Wardlaw et al., 2013; Otsugu et al., 2017; Arora et al., 2021). Animal studies have confirmed that mices infected with *Cnm*^(+)^*Sm* exhibit a significantly more severe vascular damage as compared to those infected with *Cnm*-negative (*Cnm*^(−)^*Sm*) strains (Nomura et al., 2020). The expression of the *Cnm* gene in *S. mutans* is regulated by the environmental conditions encountered in the oral cavity or in other parts of the host body, ensuring that the bacteria can adapt to different conditions within the host and aiding in both bacterium’s persistence and virulence (Lemos et al., 2019).

From a molecular-scale perspective, the *Cnm* protein contains specific domains that facilitate the adhesion of cells to extracellular matrix proteins. Such domains include the von Willebrand factor A (vWFA) domains (Sciotti et al., 1997; Thomer et al., 2013) and the fibronectin type III domains (FN3) (Aviles-Reyes et al., 2017; Otsugu et al., 2017; Chia et al., 2000; Arora et al., 2021). Both these types of domain possess high specificity for different types of collagen, particularly type I, II, and IV (Aviles-Reyes et al., 2017; Arora et al., 2021), recognize and bind to specific sequences or structures within the collagen fibers, which enables bacteria to tightly adhere to host tissues (Miller et al., 2015), evade the immune response (Huang et al., 2022), and establish infections. More specifically, the vWFA domain is an extra-large, highly conserved, and functionally significant glycoprotein domain consisting of about 200 amino acids and adopting a Rossmann fold (i.e., a central β-sheet surrounded by *α*-helices) (Whittaker and Hynes, 2002; Singh Chouhan et al., 2019); It is involved in mediating cell adhesion, migration, and binding interactions across a broad range of biological contexts (Whittaker and Hynes, 2002). While having a role in a number of normal physiological processes, the vWFA domain is also crucial in the pathogenesis of *S. mutans* (as well as of other bacteria) (Thomer et al., 2013), which highlights its dual importance in both health and disease. The domain contains a conserved metal ion-dependent adhesion site motif, which is crucial for binding divalent cations like Mg^2+^ or Mn^2+^. These ions are essential for the adhesion function of the domain. On the other hand, the FN3 domains are less extensive and typically consist of about 90~100 amino acids. The domains adopt a β-sandwich structure, which consists of seven β-strands arranged into two antiparallel β-sheets packed against each other to create a hydrophobic core that stabilizes the domain (Koide et al., 1998). Stabilization of the overall structure is guaranteed through the action of hydrogen bonds only, since, unlike vWFA, FN3 domains lack disulfide bonds (Kulahin et al., 2008; Dalton and Lemmon, 2021). Despite the above-mentioned structural differences, FN3 domains are similar to the vWFA ones in their biochemical contributions. In *Streptococci* pathogenesis, two main roles have been recognized for the FN3 domains: (i) helping the bacteria bind to host tissues; and, (ii) providing them a path to evade immune responses (Proctor, 1987; Speziale et al., 2019). The presence of the *Cnm* gene and, accordingly, the propensity to synthesize the *Cnm* protein, varies among different *S. mutans* strains. Quantitatively understanding in a fast and non-destructive way the propensity of individual *S. mutans* isolates to harbor the *Cnm* protein could greatly contribute to reduce the risk of systemic infections, prevent the formation of cerebral microbleeds, and impede the occurrence of hemorrhagic stroke (Nakano et al., 2011; Hosoki et al., 2020; Taniguchi et al., 2022).

Recently, vibrational spectroscopic techniques have widely been applied in the characterization of bacterial species and biofilm structures at the molecular level (Sandt et al., 2007; Efeoglu and Culha, 2013; Kelestemur et al., 2018; Zhu et al., 2007). Infrared spectroscopy is a widely applied tool in bioanalytical chemistry, but its spectra are prone to interference from water bond signals, which limit their characterizations to dehydrated biofilm samples (Suci et al., 1997). On the other hand, Raman spectra are not affected by –OH water signals and can provide in nearly real time multiomic snapshots of biological cultures without needing dehydration (i.e., compatibly with bacterial life) (Rae et al., 2014). In our previous Raman spectroscopic analyses of cells, bacteria, pathogenic yeasts, and viruses, we enhanced the potential of Raman spectroscopy by exploiting machine-learning algorithms to link the collected Raman spectra to a library of spectra from elementary molecules, which gives semi-quantitative accounts for the examined molecular structures (Pezzotti, 2021; Pezzotti et al., 2021a, 2021b, 2021c; Pezzotti et al., 2022a, 2022b, 2022c, 2023c). Moreover, in a previous Raman study (Pezzotti et al., 2023b), we compared the molecular structures of *in vitro* formed biofilms in *S. mutans* and *S. sanguinis* separate cultures and their cocultures at intermediate fractional populations. In that study, upon applying deconvolutive machine-learning algorithms to analyze and compare live biofilm metabolomics, we unveiled new details regarding interspecies interactions under conditions of severe competition.

In this paper, we focus on *S. mutans* bacteria and, building upon our previous Raman findings, attempt to clarify differences in molecular structures between *Cnm*^(+)^*Sm* and *Cnm*^(−)^*Sm* strains and their respective biofilms. The main goal here is to precisely locate the molecular-scale circumstances, which promote both increased adhesion and formation of cerebral microbleeds. The gained Raman insight is set to become a guide in a successive study of clinical samples, which will be the object of a future report.

## 2 Experimental procedures

### 2.1 Bacterial strains and construction of *cnm*-knockout mutans

*Cnm*^(+)^*Sm* KSM153 and *cnm*^(−)^*Sm* KSM153 *Δcnm* strains were cultured in trypticase soy broth (Becton Dickinson Microbiology Systems, Cockeysville, MD, USA) or Trypticase soy agar (TSA) added with erythromycin (10 μg/mL for KSM153 *Δcnm*) in 5% CO_2_ atmosphere at 37 °C, since *S. mutans* optimal growth requires a facultative anaerobic atmosphere (Chismirina et al., 2023). *S. mutans* KSM153 was obtained in a previous study (Watanabe et al., 2021). *Cnm* single-deletion mutants (KSM153 *Δcnm*) were also constructed via overlapping extension polymerase chain reaction (PCR), as described in detail in a previous paper (Kawada-Matsuo et al., 2013). For Readers’ convenience, a brief description of this procedure is offered hereafter. The strategy adopted to produce the *cnm* deletion mutant consisted in generating two fragments corresponding to approximately 500 bp of the upstream and downstream sequences of the gene via PCR, using KOD Plus (Toyobo, Tokyo, Japan): primer pairs for the upstream fragment *cnm*-F1 (forward 5′-CCGTTGCCATCATTTTGC-3′ and reverse 5′-CAGTCGAGGATTGCCGCCGCTGGTAA-3′) and for the downstream fragment *cnm*-F2 (forward 5′-GCT GACCTAGTAGAAGCTCCAACAACAAC-3′ and reverse 5′-GTCCCTTGGTACCAGC-3′) (Kawada-Matsuo et al., 2013). Each of the F1 reverse and F2 forward primers contained 11 bases complementary to the erythromycin resistance cassette (Em^r^), which were cloned using pBlueScript-Em (StrataGene, La Jolla, CA, USA). The Em^r^ gene was amplified via PCR using primers for Em^r^. All PCR amplicons were purified using a PCR purification kit (Qiagen KK, Tokyo, Japan). The corresponding upstream and downstream amplicons were mixed at a 1:1:1 ratio with the Em^r^ PCR product. The amplicon mixture was then used as the template for a second PCR using forward *cnm*-F1 and re- verse *cnm*-F2 primers. The resulting PCR products were finally transformed into *S. mutans* and the mutation verified using PCR (Kawada-Matsuo et al., 2013).

### 2.2 Crystal violet staining assay

Inhibition of biofilm formation was investigated by measuring the biomass of biofilms via crystal violet staining assay. First, 100 μL of tris-buffered saline (TSB) supplemented with 2% glucose and various concentrations of bacteriocin was added to the bacterial wells, then bacterial cultures (10_5_ cells) were inoculated into each well and incubated at 37 °C for 24 h. Biofilms were rinsed with phosphate-buffered saline (PBS) three times and stained with 0.1% crystal violet solution for 10 min. After rinsing with PBS to remove excess dye and successive air-drying, the stained biofilms were solubilized in 100 μL of 33% acetic acid for 15 min and the optical density (OD) determined at OD_570 nm_ with an iMark Microplate reader (Bio-Rad Laboratories, Hercules, CA, USA).

### 2.3 Raman spectroscopy and machine learning algorithms

High spectrally resolved Raman spectra were collected *in situ* on bacterial cultures using a spectrometer specially designed for measurements on biological samples (LabRAM HR800, Horiba/Jobin-Yvon, Kyoto, Japan). An optical circuit set in confocal mode with a 20x objective lens and employing a holographic notch filter concurrently enabled high signal efficiency and high spectral resolution. The wavelength of the incoming light was 532 nm, as generated by a solid-state laser source operating at 10 mW. The Raman scattered light was monitored by means of a single monochromator interfaced with an air-cooled charge-coupled device (CCD) detector (Andor DV420-OE322; 1024 × 256 pixels). The acquisition time for a single spectrum was typically 10 s for three successive acquisitions at each location. A spectral resolution of better than 1 cm^−1^ was achieved by concurrently collecting (at each measurement) an internal reference signal from a selected neon lamp to calibrate the spectrometer. Twenty spectra collected at different locations (over a total area of ~2 mm^2^) were obtained for each bacterial culture/biofilm and averaged in order to obtain representative spectra. Raman spectra were recorded on of *Cnm*^(+)^*Sm* and *Cnm*^(−)^*Sm* strains cultured with or without sucrose addition under exactly the same conditions.

Experimental Raman spectra were subjected to polynomial baseline subtraction and deconvolution into a series of Gaussian-Lorentzian band components. The baseline subtraction procedure was performed using options available in commercial software (LabSpec 4.02, Horiba/Jobin-Yvon, Kyoto, Japan) with fixed criteria for all collected spectra. All spectra were analyzed after intensity normalization to the strongest signal in the collected spectral interval. Detailed descriptions of spectral deconvolution criteria have been reported in previously published papers (Pezzotti, 2021; Pezzotti et al., 2021c). An automatic solver exploiting a linear polynomial expression of Gaussian-Lorentzian functions was iteratively run to match average experimental spectra for minimum scatter (better than 95% confidence interval) (Pezzotti, 2021).

## 3 Experimental results

### 3.1 Microscopic and biological characterizations

**Figure 1:**
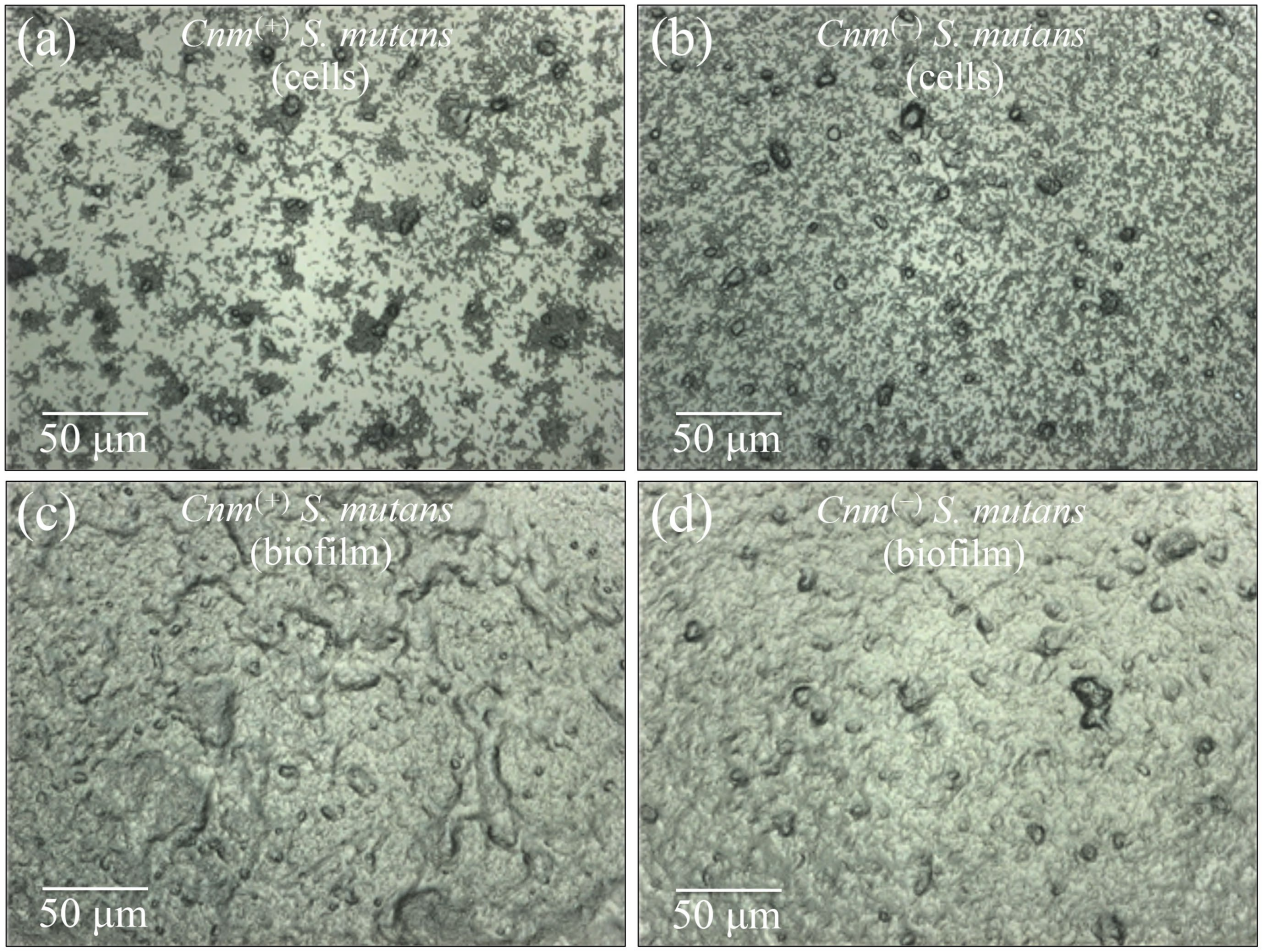
Laser micrographs of *Cnm*^(+)^*Sm* **(a)** and *Cnm*^(−)^*Sm* **(b)** cells cultured without sucrose addition. Biofilm structures clearly developed when *Cnm*^(+)^*Sm* **(c)** and *Cnm*^(−)^*Sm* **(d)** cells were cultured with addition of sucrose.

Figures 1a,b show laser micrographs of *Cnm*^(+)^*Sm* and *Cnm*^(−)^*Sm* strains, respectively, cultured without addition of sucrose. In such culture conditions, bacteria cannot produce biofilm. The two micrographs show one important detail by which the two cultures differed: the *Cnm*^(+)^*Sm* bacterial cells (a) tended to group more easily than the *Cnm*^(−)^*Sm* ones (b) [cf. the higher number and bigger size of cell agglomeration in (a) as compared to (b)]. This important observation points to a different capacity of adhesion between the two strains and, thus, suggests a structural difference at the molecular level in cell surface composition. This point will be confirmed in the Raman analyses shown in the following sections. Figures 1c,d show laser micrographs of *Cnm*^(+)^*Sm* and *Cnm*^(−)^*Sm* strains, respectively, cultured with addition of sucrose. As seen, in both cultures, the presence of bacterial cells is hardly resolved, while only massive biofilm volumes are commonly envisaged. At a glance, the *Cnm*^(+)^*Sm* strain seemed to produce more abundant amounts of biofilm under the same culture conditions. This point was confirmed by measuring the biomass of biofilms via crystal violet staining assay. The result of this assay showed that the *Cnm*^(+)^*Sm* strain produced 10 ± 2% higher amount of biofilm than the *Cnm*^(−)^*Sm* one under exactly the same culture conditions. The Raman results shown later will shed light on possible compositional differences.

### 3.2 Raman spectroscopic assessments

**Figure 2:**
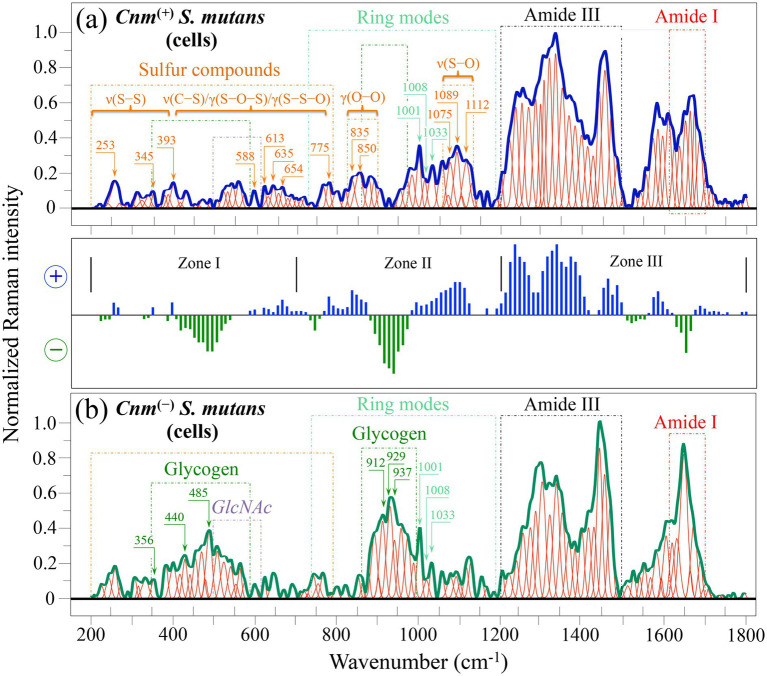
Average Raman spectra of *Cnm*^(+)^*Sm* **(a)** and *Cnm*^(−)^*Sm* **(b)** cells cultured without sucrose in the wavenumber interval 200–1800 cm^−1^. The intermediate plot, which represents the difference in spectral intensity **(a,b)**, is divided into three characteristic spectral zones (cf. labels in inset). The main molecular features discussed in the text are emphasized in inset to each spectrum. Wavenumbers are in cm^−1^.

Figure 2 shows Raman spectra collected in the wavenumber interval 200~1800 cm^−1^ on (a) *Cnm*^(+)^*Sm* and (b) *Cnm*^(−)^*Sm* strains cultured in absence of sucrose. Each spectrum is the average of 20 spectra collected at different locations (over a total area of ~2 mm^2^). Since in absence of sucrose both cultures lack biofilm (cf. Figures 1a,b), the spectra represent the molecular structure of bacterial cells only. The graph displayed between spectra in (a) and (b) represents a difference plot of Raman intensities as a function of wavenumber, which was obtained by subtracting the spectrum of the *Cnm*^(−)^*Sm* strain [in (b)] from that of the *Cnm*^(+)^*Sm* one [in (a)]. This latter plot helps to better figure out the spectral differences between the two strains. The spectra, which were deconvoluted into a series of Gaussian-Lorentzian sub-bands, were divided into three main wavenumber sub-zones, henceforth referred to as Zones I, II, and III in the intervals 200~700, 700~1200, and 1200~1800 cm^−1^, respectively. As can be seen, the spectral morphology in *Cnm*^(+)^*Sm* and *Cnm*^(−)^*Sm* strains is very different over the entire wavenumber span investigated. Such differences are unequivocal proofs for a significant divergence in molecular structure between the two strains. In inset to each spectrum, the main differences are emphasized with reference to spectral signals related to specific molecules of interest. In particular, Zones I and II are rich in signals from sulfur-containing molecules in the spectrum of the *Cnm*^(+)^*Sm* strain (a), which conspicuously lack in the spectrum of the *Cnm*^(−)^*Sm* strain (b). On the other hand, signals characteristic of glycogen molecules are clearly stronger in the spectrum of the *Cnm*^(−)^*Sm* strain (b), but conspicuously missing in that of the *Cnm*^(+)^*Sm* strain (a). In Zone II, ring modes from phenylalanine appeared stronger in the spectrum in (a). An additional striking feature was the predominance of signals in Zone III (comprising the Amide III zone of proteins) in the spectrum of the *Cnm*^(+)^*Sm* strain (a), while the Amide I zone (also related to vibrational modes of proteins in the amide plane) at higher wavenumber only partly followed the same pattern. A detailed analysis of such spectral differences will be given in the next section.

**Figure 3:**
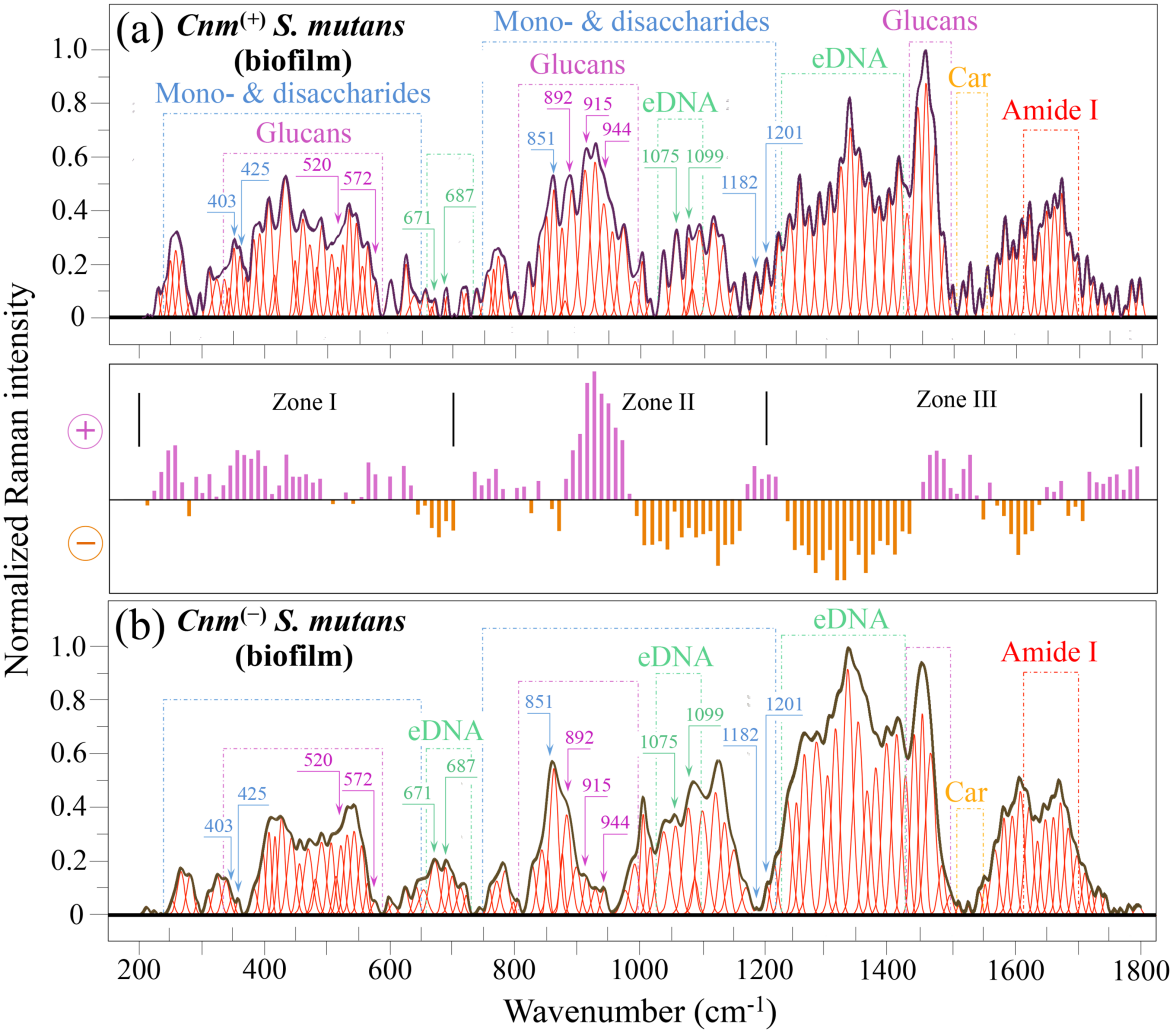
Average Raman spectra of *Cnm*^(+)^*Sm* **(a)** and *Cnm*^(−)^*Sm* **(b)** cells cultured with sucrose in the wavenumber interval 200–1800 cm^−1^. The spectra mainly represent biofilm structures (cf. Figures 1c,d). The intermediate plot, which represents the difference in spectral intensity **(a,b)**, is divided into three characteristic spectral zones (cf. labels in inset). The main molecular features discussed in the text are emphasized in inset to each spectrum. Wavenumbers are in cm^−1^.

Figure 3 shows Raman spectra collected in the wavenumber interval 200~1800 cm^−1^ on (a) *Cnm*^(+)^*Sm* and (b) *Cnm*^(−)^*Sm* strains cultured with addition of sucrose. Similar to the spectra in Figure 2, each spectrum was the average of 20 spectra collected at different locations (over a total area of ~2 mm^2^). Both spectra could be considered to mainly represent the molecular structure of the respective bacterial biofilms, as suggested by the micrographs in Figures 1c,d. The intermediate graph between spectra in (a) and (b) represents a difference plot of Raman intensities as a function of wavenumber, which was obtained by subtracting the spectrum of the *Cnm*^(−)^*Sm* biofilm (in (b)) from that of the *Cnm*^(+)^*Sm* one [in (a)]. Similar to spectra shown in Figure 2, the spectra in Figure 3 were deconvoluted into a series of Gaussian-Lorentzian sub-bands and divided into Zones I, II, and III in the intervals 200~700, 700~1200, and 1200~1800 cm^−1^, respectively. Also in the case of biofilm, the morphology of spectra from *Cnm*^(+)^*Sm* and *Cnm*^(−)^*Sm* strains was very different over the entire wavenumber span investigated. Such difference hints at quite different metabolic strategies and priorities selected by the two strains. In inset to each spectrum, the main differences are emphasized with reference to spectral signals related to specific molecules of interest. The spectrum of *Cnm*^(+)^*Sm* biofilm was clearly richer in signals from glucans and mono/disaccharides (Zones I and II), while Zone III hinted to a higher amount of extracellular DNA in the spectrum of the *Cnm*^(−)^*Sm* biofilm (b). A more detailed analysis of such spectral differences in biofilm structure given in Section 3.4.

### 3.3 Raman analyses of bacterial cell structures

Despite being the major causative pathogen for dental caries, *S. mutans* is incapable of binding to soft tissues with the exception of strains containing the *Cnm* gene (i.e., reported as ~10% of the total clinical isolates of *S. mutans*) (Sato et al., 2004). The role of *Cnm* in pathogenicity of *S. mutans* has extensively been investigated and the biological properties of *Cnm* thoroughly elucidated with emphasis on genomic aspects and related modifications (Ajdic et al., 2002; Maruyama et al., 2009; Aviles-Reyes et al., 2014a). Specifically regarding structural analyses, Nomura et al. (2013) succeeded in characterizing the *Cnm* motif-anchored protein of *S. mutans* as a novel LPXTG (namely, leucine, proline, X, threonine, and glycine, with X being any amino acid). The following Raman analyses aims at unfolding yet unreported molecular-scale details about *Cnm*^(+)^*Sm* and *Cnm*^(−)^*Sm* strains and their respective biofilms, which are likely elusive to conventional analyses of bacterial structure.

High-resolution electron microscopy images of *Cnm*^(+)^*Sm* and *Cnm*^(−)^*Sm* strains, as reported by Naka et al. (2022), revealed bumpy structures observed on the surfaces of *Cnm*^(+)^*Sm* cells and suggested that *Cnm* stems as a cell-surface protein. This could be considered as a plausible hypothesis given the absence of any pilus or fimbrial structure in gram-positive bacteria and the phenomenologically proven roles of the *Cnm* protein in *S. mutans* cells, when they both enter in contact with environmental niches of bacteria/host adhesion and escape from host defense systems (Bucior et al., 2012; Chagnot et al., 2013). In the present study, we show that deletion of *Cnm*–related genomes resulted in dramatic changes of the molecular structure for the bacterial cells; the details of such changes are discussed in detail hereafter for specific spectral regions with focusing on spectral bands peculiarly observed in *Cnm*^(+)^*Sm* or *Cnm*^(−)^*Sm* strains.

#### 3.3.1 Zone I—low wavenumber region (200~700 cm^−1^)

**Figure 4:**
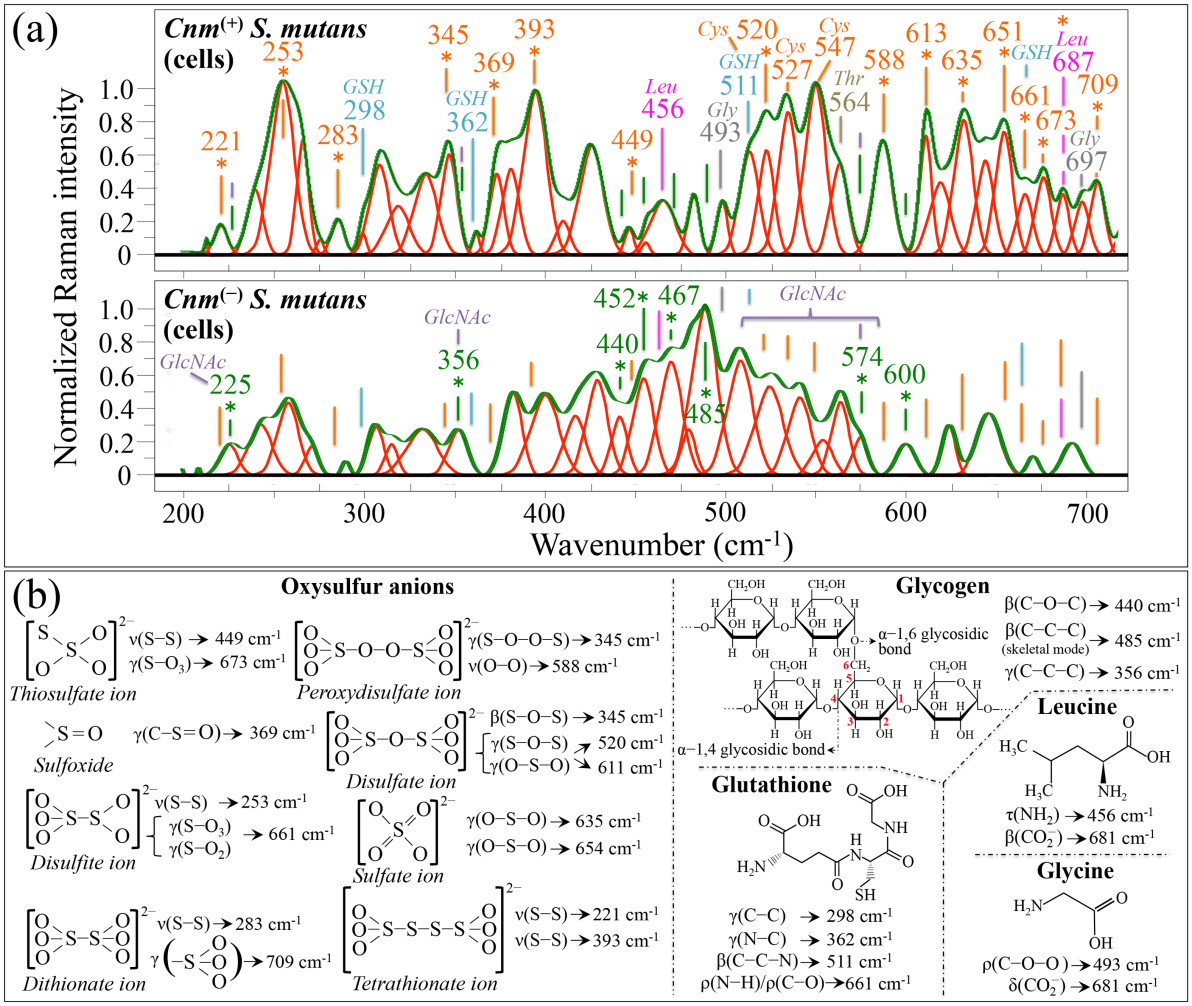
**(a)** Average Raman spectra of *Cnm*^(+)^*Sm* (upper) and *Cnm*^(−)^*Sm* (lower) cells cultured without sucrose in the wavenumber interval 200–700 cm^−1^ (Zone I). Both spectra are deconvoluted into Lorentzian-Gaussian bands (cf. band wavenumbers in inset). The main molecular features discussed in the text are emphasized in inset to each spectrum (abbreviations: *GSH* ➔ glutathione, *Leu* ➔ leucine, *Gly* ➔ glycine, and *GlcNAc* ➔ *N*-acetyl-D-glucosamine). In **(b)**, vibrational origins are shown for signals belonging to oxysulfur anions, glycogen, glutathione, leucine, and glycine (symbols: *ν* ➔ stretching, β ➔ bending, *γ* ➔ deformation, *ρ* ➔ rocking, and *τ* ➔ twisting). Wavenumbers are in cm^−1^.

As previously shown in Figure 2, this spectral zone included profound morphological differences between the Raman spectra of *Cnm*^(+)^*Sm* and *Cnm*^(−)^*Sm* strains. In our spectroscopic analysis, we tracked down 25 Raman signal (over al total of 39) in the spectrum of *Cnm*^(+)^*Sm* strain, which could not be found (or were significantly weaker) in the spectrum of the *Cnm*^(−)^*Sm* strain (cf. labeled sub-bands in the upper spectrum of Figure 4a; cf. labels in inset). This datum alone give evidence of how different were the spectra collected on *Cnm*^(+)^*Sm* and *Cnm*^(−)^*Sm* strains. The presence of *Cnm* strongly enhanced the cysteine band located at 527 cm^−1^, which represents the S–S stretching mode in this amino acid structure (i.e., disulfide bonds between two cysteine residues in proteins) (Diaz Fleming et al., 2009). Note also that cysteine S–S stretching also contributes two additional signals at 511 and 547 cm^−1^; however, the former signal overlaps with a strong scatter from glutathione, as discussed later. Among the remaining 22 signals missing in the spectrum of the *Cnm*^(−)^*Sm* strain, 15 signals (labeled with a orange asterisk in Figure 4a) could be tracked back to oxysulfur anions (cf. structures and vibrational origins in Figure 4b) (Sato et al., 1985). These band components, which in the *Cnm*^(+)^*Sm* strain appear to represent a complex mixture, are likely both by-products and decomposition products in the metabolic cycle of *S. mutans*. A striking feature in the spectrum of the *Cnm*^(+)^*Sm* strain was found in the strong band component at 253 cm^−1^, which arises from S–S stretching in disulfide ions (cf. Figures 4a,b). Since both *Cnm*^(+)^*Sm* and *Cnm*^(−)^*Sm* strains were treated and analyzed under exactly the same conditions, these important spectroscopic findings hint to a significant inhibition of methionine sulfoxide reductases (Msrs) in the *Cnm*^(+)^*Sm* strain (Singh et al., 2018). Msrs indeed repair proteins from oxidative damage by eliminating sulfoxide residues, and play a fundamental role in protein regulation and cell survival (Lee and Gladyshev, 2011). We interpret the interruption in producing Msrs proteins by *Cnm*^(+)^*Sm* bacteria as a strategy to increase their adhesion capacity (this point will find a confirmation in the analysis of Zone II shown later). This interpretation is in line with the microscopy observations by Naka et al. (2022), but further suggests that residual oxysulfur molecules were delivered to the bumpy structures covering the surface of *Cnm*^(+)^*Sm* cells. In other words, *Cnm*^(+)^*Sm* bacteria recycle (rather than eliminate) the “garbage” (sulfur oxidative products) in order to reuse it for anchoring purposes. Note also that two of the strongly enhanced signals related to sulfur compounds found in the spectrum of *Cnm*^(+)^*Sm* bacteria, namely, those at 635 and 651 cm^−1^ (cf. Figure 4a), are likely contributed by indoxyl sulfate molecules (Elumalai et al., 2015). This point will be confirmed and discussed in later sections.

In a recent paper (Pezzotti et al., 2023a), we reported another case in which another oral bacteria, the gram-negative *Porphyromonas gingivalis*, reused its own oxysulfur molecules for a specific purpose. This bacterium introduces such molecules into ceramide-enveloped outer-membrane vesicles and then shoots them against antagonist cells and bacteria. *In situ* Raman screening of low-concentration (i.e., in the order of 10 μg/mL) vesicle-exposed neuroblastoma cells revealed a complete aggregation of the cells into amyloid-*β* clusters in times as short as 6 h. In analyzing exosomes from *Porphyromonas gingivalis*, a low-wavenumber Raman spectroscopic scenario with strong metabolic fingerprints for disulfide-linked multimers (in comparison to a control culture) could be revealed. This proves how, in analogy with the present case, the presence of oxysulfur compounds is key in enhancing protein anchoring. In the case of *Cnm*^(+)^*Sm* cells, sulfur-recycling might be exploited for promoting adhesion and successive accumulation in arterial and venous enclaves. Despite obviously different chemical details, exploiting the stickiness of sulfuric compounds appears to be a common strategy adopted by oral pathogens in a number of different purposes.

Among signals present in the *Cnm*^(+)^*Sm* strain, but absent in the *Cnm*^(−)^*Sm* one, two bands (at 456 and 687 cm^−1^) can be tracked back to leucine (cf. structure and vibrational origins in Figure 4b) (Kumar, 2011), although the latter might also contain strong contributions from indoxyl sulfate (Elumalai et al., 2015). Leucine is indeed one of the amino acid residues present in the motif-anchored *Cnm* protein (Nomura et al., 2013). Two more bands at 493 and 697 cm^−1^ are mainly contributed by glycine and one at 564 cm^−1^ by threonine (Rolinski et al., 2014), which are also main amino acid component of the *Cnm* motif-anchored protein (cf. reference spectra from *Cnm* amino acids collected in Supplementary Figure S1) (Nomura et al., 2013). Two additional weak but clearly discernable signals, only seen in the *Cnm*^(+)^*Sm* spectrum, could be found at 298 and 362 cm^−1^ (cf. labels in Figure 4a). These two signals are peculiar to the glutathione molecule tentatively assigned to C–C and N–C deformation, respectively; (cf. Figure 4b) (Shiota et al., 2018). Two additional strong signals from glutathione are expected at 511 and 661 cm^−1^ (assigned to C–C–N bending and N–H or C–O rocking, respectively; cf. also the spectrum of glutathione reported in Supplementary Figure S2). However, these latter two bands strongly overlap with signals from sulfur and oxysulfur compounds (cf. labels in inset to Figure 4a), and thus can hardly serve as markers for glutathione molecules. Li et al. (2020), reported that the post-translational process of S-glutathionylation, which selectively targets protein cysteine thiols, plays a crucial role in *S. mutans* under both oxidative and nitrosative conditions in interspecies competition and under immunochemical stress. The presence of bands that could only be assigned to glutathione in the *Cnm*^(+)^*Sm* spectrum definitely reflects both such activities. Note that glutathione molecules are indeed present in the trypticase soy broth used for culturing, but they stem in concentrations less than 0.5 μM (Reniere et al., 2015). Due to such a low concentration, Raman screening is incapable to detect glutathione in the *Cnm*^(−)^*Sm* sample. On the other hand, the *Cnm*^(+)^*Sm* strain appears capable to uptake glutathione molecules from the growth medium and to store them intracellularly in concentrations high enough to be detectable by the Raman probe. This hypothesis is in line with a study by Sherrill and Fahey (1998), in which some specific strains of *S. mutans* were shown capable to import and metabolize glutathione. In summary, the *Cnm*^(+)^*Sm* strain, while collecting oxysulfur compounds and using them for enhancing tissue adhesiveness, yet enhances its ability to scavenge glutathione to counteract nitrosative processes and to activate its virulence gene expression.

Looking now at the spectrum of *Cnm*^(−)^*Sm* strain, 8 bands could be located, which are conspicuously absent in the spectrum of the *Cnm*^(+)^*Sm* strain. These bands are located at 225, 356, 440, 452, 467, 485, 574, and 600 cm^−1^ (cf. labels in inset to Figure 4a lower spectrum) and can be assigned to mono and polysaccharides (Konorov et al., 2011; Wiercigroch et al., 2017). Five bands in the narrow interval 350 ~ 600 cm^−1^, namely, at 356, 440, 485, 574, and 600 cm^−1^, are strongly contributed by glycogen molecules (cf. vibrational origins in Figure 4b). In particular, the signal at 485 cm^−1^ has been reported as a specific marker for glycogen, because it is essentially free of overlapping Raman contributions from other molecules in the bacterial structure (i.e., amino acids and DNA) (Konorov et al., 2011). Note also that the two bands at 225, 356, and the signal at 574 cm^−1^ (with the overall spectral zone 500~600 cm^−1^) are preponderant signals in the spectra of both D-glucosamine and N-acetyl-D-glucosamine (*GlcNAc*) (She et al., 1974), which are important constituents of the peptidoglycan structure of *S. mutans*. This point will be discussed later. The signals at 225 and 467 cm^−1^ include contributions from D–(+)–glucose and D–(+)–sucrose, respectively (Wiercigroch et al., 2017). The above spectral scenario unveil a superior ability for the *Cnm*^(−)^*Sm* strain to produce and store glycogen as compared to the *Cnm*^(+)^*Sm* one. This suggests that the genes involved in glycogen metabolism might be downregulated or differently regulated in the *Cnm*^(+)^*Sm* strain, possibly as an adaptive response to their niche or due to enhanced virulence factors. Specific mutations in the genes encoding enzymes responsible for glycogen synthesis, such as glycogen synthase, impair this function. *Cnm*^(+)^*Sm* might allocate its resources differently, focusing on virulence factors of the *Cnm* protein rather than glycogen storage, which could be an adaptive advantage in tissue invasive activity. This interpretation is also consistent with a possible simplification of the peptidoglycan structure in the *Cnm*^(+)^*Sm* structure (i.e., cf. trend for *GlcNAc* bands): if oxysulfur compounds-rich *Cnm* proteins wrap the bacterial cell, the protective function of the peptidoglycan layer becomes less critical and its production could be minimized.

#### 3.3.2 Zone II—intermediate wavenumber region (700~1200 cm^−1^)

**Figure 5:**
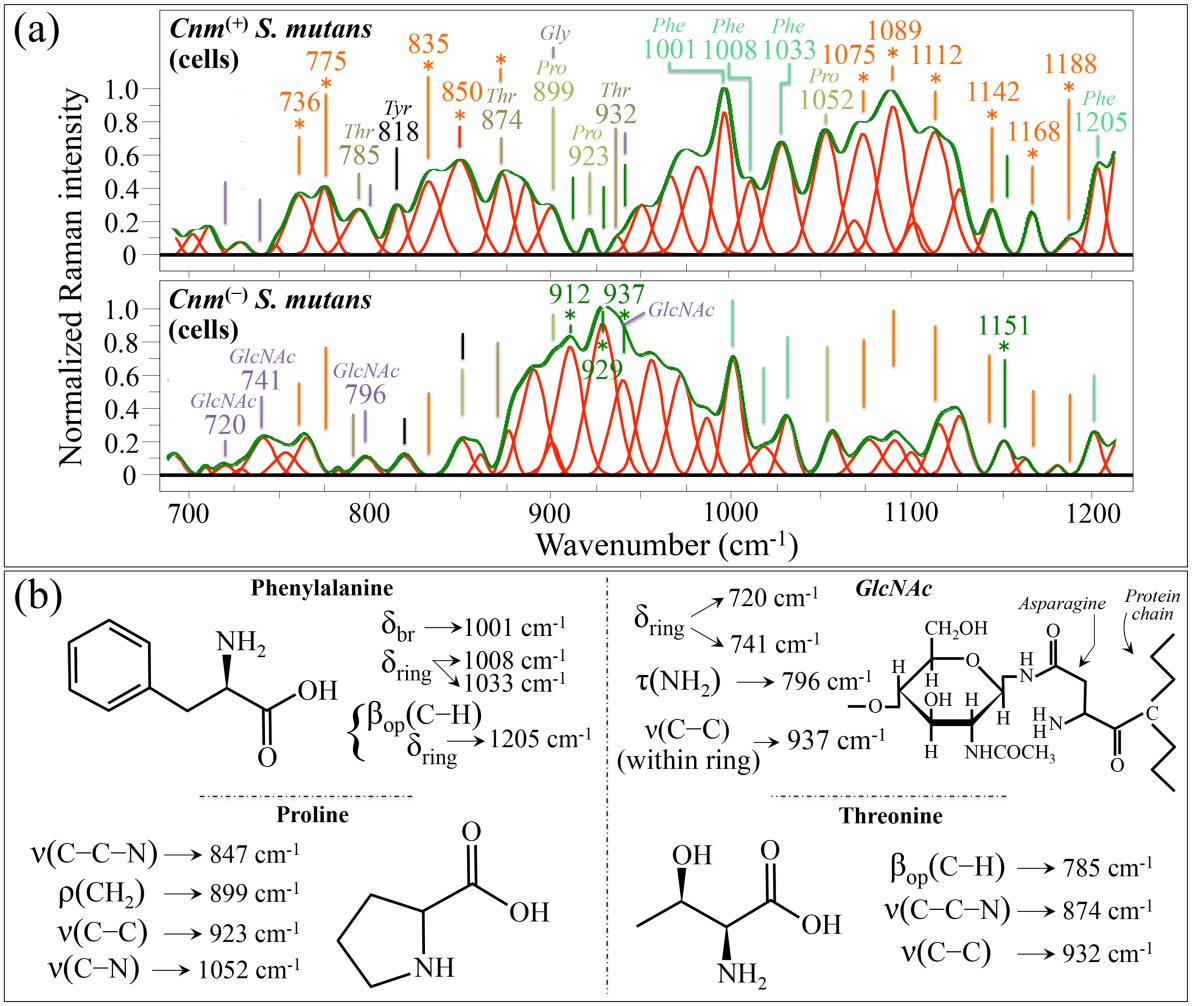
**(a)** Average Raman spectra of *Cnm*^(+)^*Sm* (upper) and *Cnm*^(−)^*Sm* (lower) cells cultured without sucrose in the wavenumber interval 700–1200 cm^−1^ (Zone II). Both spectra are deconvoluted into Lorentzian-Gaussian bands (cf. band wavenumbers in inset). The main molecular features discussed in the text are emphasized in inset to each spectrum (abbreviations: *Thr* ➔ threonine, *Tyr* ➔ tyrosine, *Leu* ➔ leucine, *Pro* ➔ proline, *Gly* ➔ glycine, *Phe* ➔ phenylalanine, and *GlcNAc* ➔ *N*-acetyl-D-glucosamine). In **(b)**, vibrational origins are shown for signals belonging to phenylalanine, proline, *GlcNAc*, and threonine (symbols: ν ➔ stretching, β_op_ ➔ out-of-plane bending, δ_br_ ➔ ring breathing, δ_ring_ ➔ deformation, ρ ➔ rocking, and τ ➔ twisting). Wavenumbers are in cm^−1^.

Looking at the plot of spectral differences in Figure 2, one could notice a preponderance in Raman signals in the spectrum of the *Cnm*^(+)^*Sm* strain above that of the *Cnm*^(−)^*Sm* (blue plot) within two main wavenumber intervals: 770~880 cm^−1^ and 990~1190 cm^−1^. In the former zone, in addition to a significantly enhanced signal at 850 cm^−1^, two signals at 775 and 835 cm^−1^ appear to be peculiar to the *Cnm*^(+)^*Sm* strain (Figure 5). The presence of these two latter signals (O–O deformation) (Diaz Fleming et al., 2009) confirms the accumulation of oxysulfur molecules already noticed in Zone I, while the strongly enhanced intensities at 736 and 850 cm^−1^ in the *Cnm*^(+)^*Sm* strain are attributable to indoxyl sulfate (cf. Figure 5a) (Elumalai et al., 2015). Note, however that the enhancement of the signal at 850 cm^−1^ could also incorporate contributions from proline C–C–N stretching (cf. Figure 5b) and leucine (C–C=O stretching) (Kumar, 2011; Zhu et al., 2011), which are two amino acid components of the *Cnm* protein (Nomura et al., 2013). On the other hand, increases of three main signals from proline residues at 899, 923, and 1052 cm^−1^ which could tentatively be assigned to CH_2_ rocking, C–C stretching, and C–N stretching, respectively (cf. Figure 5b) (Zhu et al., 2011). The proline band at 899 cm^−1^ is also contributed by a strong signal from glycine, while the three bands at 785 (C–H out-of-plane bending), 874 (C–C–N stretching), and 932 cm^−1^ (C–C stretching; cf. Figure 5b), which are conspicuously absent in the *Cnm*^(−)^*Sm* spectrum, could be assigned to threonine residues (cf. Figure 5b) (Zhu et al., 2011). Note that signals related to all the main amino acid residues present in the *Cnm* motif-anchored protein appeared with enhanced intensity in the spectrum of the *Cnm*^(+)^*Sm* strain (cf. also Supplementary Figure S1). Four phenylalanine bands at 1001, 1008, 1033, and 1205 cm^−1^ (ring breathing, ring deformation, ring deformation, out-of-plane C–H bending/ring deformation, respectively; cf. Figure 5b) (She et al., 1974) also appeared more intense in the Raman spectrum of the *Cnm*^(+)^*Sm* strain. The origin of such enhancement will be further discussed in later sections. In the ring zone, two main bands can be assigned to ring vibrations in tyrosine residues (Zhu et al., 2011), although they strongly overlap with signals from other compounds (cf. Figure 5a). In the 990 ~ 1190 cm^−1^ wavenumber interval, three additional strong signals, observed in the spectrum of the *Cnm*^(+)^*Sm* strain but conspicuously absent in that of the *Cnm*^(−)^*Sm* one, were found at 1075, 1089, and 1112 cm^−1^ (cf. Figure 5). These three bands can be assigned to S–O stretching in oxysulfur anions (Sato et al., 1985). Three additional bands at 1142, 1168, and 1188 cm^−1^, only observed in the spectrum of the *Cnm*^(+)^*Sm* strain, also point to S–O stretching vibrations in sulfur compounds with higher oxygen stoichiometry (Sato et al., 1985). Overall, the signal enhancement zone in the interval 990~1190 cm^−1^ confirms the interpretation of signal enhancements due to oxidized sulfur compounds given for Zone I (cf. Figure 4).

Looking now at the spectral zone in which the *Cnm*^(−)^*Sm* spectrum is richer in Raman scattering (i.e., the 880~990 cm^−1^ interval shown in green color in the difference plot of Figure 2), the most prominent signals are seen at 912, 929, and 937 cm^−1^. These bands represent C–C stretching in glucose and glycogen molecules (Konorov et al., 2011). This strong signal enhancement confirms the hypothesis of downregulation for the genes involved in glycogen metabolism in the *Cnm*^(+)^*Sm* strain, in line with a trend suggested by signal characteristics in Zone I. A similar interpretation could be given for the (weaker) signal seen at 1151 cm^−1^ (C–O and C–C stretching in glucose) (Konorov et al., 2011), which is missing in the *Cnm*^(+)^*Sm* spectrum (cf. Figure 5). Finally, three additional signals at 720, 741, and 796 cm^−1^ (for vibrational assignments cf. Figure 5b) were clearly seen in the *Cnm*^(−)^*Sm* spectrum but were conspicuously absent in the *Cnm*^(+)^*Sm* one (cf. labels in inset to Figure 5). These bands do not belong to the vibrational characteristics of glucose or glycogen; they could instead be assigned to *GlcNAc* molecules (She et al., 1974). Note that these latter molecules were already found to significantly contribute signals in Zone I (at 225, 356, and 574 cm^−1^; cf. labels in Figure 4) and are only seen in the spectrum of the *Cnm*^(−)^*Sm* strain. Unfortunately stronger signals from *GlcNAc* molecules at 937 and 973 cm^−1^ (She et al., 1974; De Gelder et al., 2007), overlap with glycogen and other glycans contributions (cf. above) and cannot univocally be assigned to bacterial membrane components. As previously stated, *GlcNAc* is a major component of membrane peptidoglycans in *S. mutans*, and the conspicuous weakening of *GlcNAc* signals in the *Cnm*^(+)^*Sm* spectrum is a key finding in this study. Naka et al. (2022), observed bumpy protein structures on the surfaces of *Cnm*^(+)^*Sm* cells and suggested that the *Cnm* proteins cover the bacterial membrane. The present Raman data newly reveals that such coating concurrently involves a simplification of membrane the peptidoglycan structure in the *Cnm*^(+)^*Sm* strain. Glycosylation plays a critical role in post-translational modifications that modulate the bacterial traits associated with a number of functions including adhesion and evasion of the immune system (Valguarnera et al., 2016; Lin et al., 2020; Bhat et al., 2019). Since the spectrum of *Cnm*^(+)^*Sm* cells significantly weakened *GlcNAc* signals, it is suggested that this strain adopts a completely different strategy in cell protection and adhesion. Finally, note that the present data are in line with a recent study by di Mojana Cologna et al. (2023), in which evidence was provided for *S. mutans* bacteria being capable to switch to a completely different adhesion mechanism (i.e., heavy phosphorylation of *Cnm*) upon inactivation of pgfS glycosyltransferase.

#### 3.3.3 Zone III—high wavenumber region (1200~1700 cm^−1^)

**Figure 6:**
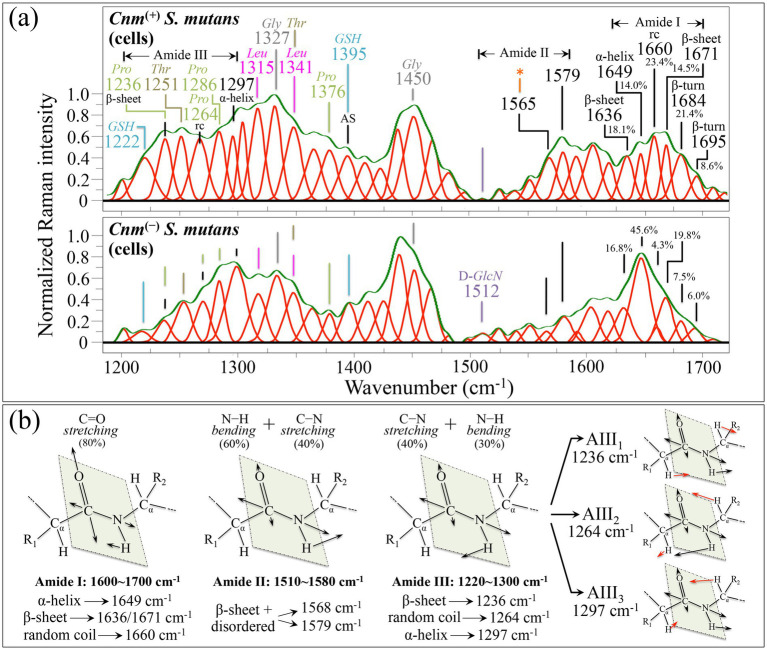
**(a)** Average Raman spectra of *Cnm*^(+)^*Sm* (upper) and *Cnm*^(−)^*Sm* (lower) cells cultured without sucrose in the wavenumber interval 1200–1700 cm^−1^ (zone III). Both spectra are deconvoluted into Lorentzian-Gaussian bands (cf. band wavenumbers in inset). The main molecular features discussed in the text are emphasized in inset to each spectrum (*rc*➔ random coil and AS➔ component of Amide III; the other abbreviations are the same as those given in Figures 4, 5). **(b)** Explanatory drafts are shown for amides I, II, and III vibrational modes in different protein secondary structures. Wavenumbers are in cm^−1^.

In the diagram of spectral differences between *Cnm*^(+)^*Sm* and *Cnm*^(−)^*Sm* spectra in Figure 2, the wavenumber zone between 1200 and 1500 cm^−1^ appears strongly enhanced in the *Cnm*^(+)^*Sm* spectrum (cf. blue plot). The interval 1220~1300 cm^−1^, which includes the main signals from the so-called Amide III zone, is protein structure-sensitive and its intensity enhancement likely arises from the presence of *Cnm* protein residues on the bacterial surface. Moreover, this wavenumber interval includes CH_2_ wagging vibrations from glycine backbone and proline side chain (cf. labels in inset to Figure 6a) in addition to C–H deformation signals from proteins (Talaikis et al., 2020). The Amide III mode, which includes in-phase C–N stretching vibration coupled with N–H in-plane bending (cf. schematic draft in Figure 6b), is located within a quite complex assembly of spectral bands. Its signal composition comprehends contributions from the so-called AIII_3_, AIII_2_, and AIII_1_ modes (reported at around 1231, 1264, and 1292 cm^−1^, respectively, for proteins in zwitterionic state), which involve different fractional contributions from NH_2_ wagging, N–H in-plane bending, and C–N stretching, in partial overlap with C–H in-plane bending (Wang et al., 1991; Schweitzer-Stenner et al., 2002). According to Schweitzer-Stenner et al. (2002), the difference between the above three modes mainly relates to the mixing with the C–H bending mode. The AIII_3_ involves an out-of-phase mixing with in-plane and out-of-plane C–H bending from the R1 side (cf. Figure 6b) in addition to an in-phase mixing with in-plane C–H bending from the R2 side. On the other hand, AIII_2_ consists of an in-phase combination between N–H in-plane bending and in-plane C–H bending from the R1 side, while the AIII_1_ signal represents contributions from all C–H bending modes. AIII_1_ and AIII_2_ signals also include NH_2_ wagging vibrations (cf. Figure 6b). AIII_3_, AIII_2_, and AIII_1_ modes tend to shift towards higher wavenumbers with decreasing pH. In the present experiments, bands representing the three modes of Amide III in the spectra of *Cnm*^(+)^*Sm* and *Cnm*^(−)^*Sm* strains differed mostly with respect to the relative intensities of the observed bands rather than in their wavenumber shifts. In both strains, the AIII_3_, AIII_2_, and AIII_1_ bands were observed at around 1236, 1264, and 1297 cm^−1^, suggesting that, independent of the presence of a layer of *Cnm* proteins on the bacterial surface, the *Cnm*^(+)^*Sm* and *Cnm*^(−)^*Sm* strains similarly preserve an acidic surface pH in agreement with the published literature (Bowden and Hamilton, 1987; Lemos et al., 2019). Note that the AIII_3_ and AIII_2_ bands partly overlap with the two lower wavenumber bands of the characteristic C–H in-plane bending triplet of proline residues (cf. Supplementary Figure S2), while the third band from the triplet is located at 1286 cm^−1^ and includes a lesser contribution from leucine (Zhu et al., 2011; Rolinski et al., 2014). Besides the overall higher Raman intensity of the *Cnm*^(+)^*Sm* spectrum in the Amide III zone, which clearly arises from a higher content of proteins specifically containing proline and threonine residues (cf. labels in inset to Figure 6a), the observed variation in relative intensity of the Amide III bands retains a specific structural meaning. As independently reported in previous publications by our and other research groups (Dehring et al., 2006; Takahashi et al., 2014), the secondary structure in proteins can be assessed in a semi-quantitative manner by computing the relative intensity ratio of the AIII_3_, AIII_2_, and AIII_1_ taken as representative of the β-sheet, random coil, and *α*-helix configurations, respectively. A higher relative intensity of the Amide III doublet, I_AIII2_/I_AIII1_ = I_1264_/I_1297_, should thus represent a higher content of disordered protein structure, which is indeed the case here for the *Cnm*^(+)^*Sm* strain with I_1264_/I_1297_ ≅ 1.7 vs. the 0.4 value measured in the *Cnm*^(−)^*Sm* strain (cf. Figure 6a). In other words, the presence of a *Cnm* envelope introduces clear vibrational fingerprints of disorder in the bacterial structure. This point will be discussed in more details later in the context of the interpretation of Amide I bands.

The zone between 1300 and 1500 cm^−1^, preponderant in the spectrum of *Cnm*^(+)^*Sm* strain, indeed includes the strongest signals from the amino acid residues involved with the *Cnm* motif-anchored protein (cf. Supplementary Figure S1). The 1315 and 1341 cm^−1^ bands, assigned to C–H rocking/deformation are fingerprint for leucine (the latter also contributed by threonine), while the band at 1327 cm^−1^ can be considered as a main marker for glycine together with the strong signal recorded at 1450 cm^−1^ (H–C–H and N–C–H rocking) (Zhu et al., 2011). An additional band, which appears clearly enhanced in the spectrum of *Cnm*^(+)^*Sm* strain, was found at 1395 cm^−1^. This bands represent a strong signal in the spectrum of glutathione (CO_2_ scissoring; cf. Supplementary Figure S2) and pairs with another prominent glutathione signal at 1222 cm^−1^ (C–N stretching). The presence of these bands confirm the hypothesis, advanced in the context of the discussion of Zone I, that the *Cnm*^(+)^*Sm* strain scavenges glutathione molecules from the growth medium in order to enhance its ability to counteract nitrosative processes and to activate virulence gene expression. Note, however, that the band at 1395 cm^−1^ has also been reported as a component of the Amide III family and referred to as AS (Wang et al., 1991). The AS component arises from C_α_–H bending vibrations and is seen only in disordered peptide structures (i.e., is completely absent in the α-helix structure). The enhanced signal at 1395 cm^−1^ thus represents another spectroscopic fingerprint for the disordered structure of the *Cnm* motif-anchored protein.

Directly related to proteins is also the wavenumber interval 1510~1580 cm^−1^, which is often referred to as the Amide II zone and is due to the vibrational coupling of out-of-phase C–N stretching and in-plane N–H bending vibrations (Di Rocco et al., 2001; Gautam et al., 2015). Similar to the Amide III zone, the Raman profile of the *Cnm*^(+)^*Sm* strain in the Amide II zone was enhanced as compared to that of the *Cnm*^(−)^*Sm* strain (cf. Figure 6a). In particular, the enhancement of the two bands at 1565 and 1579 cm^−1^ cannot be ascribed to contributions from signals of amino acid residues in the *Cnm* motif-anchored protein (cf. Supplementary Figure S2). Talaikis et al. (2020) reported a considerable enhancement of the Amide II bands in Raman spectra of proteins excited in the visible spectral region due to aggregation of disordered peptides into the β-sheet secondary structure. In the following, we shall show that this is indeed a plausible scenario for the case of *Cnm*^(+)^*Sm* strain. However, the band at 1565 cm^−1^ is likely contributed by a signal from indoxyl sulfate (Elumalai et al., 2015), as already reported in the contexts of both Zones I and II.

Curve fitting from the Raman profiles of both *Cnm*^(+)^*Sm* and *Cnm*^(−)^*Sm* strains showed the presence of completely different components in the Amide I region (stemming approximately within the wavenumber interval 1600~1700 cm^−1^; cf. Figures 6a,b). According to published literature (Krimm and Bandekar, 1986; Dong et al., 1990; Sadat and Joye, 2020), the bands at 1649 and 1660 cm^−1^ were attributed to *α*-helix and random coil structures, respectively. The bands at 1636 and 1671 cm^−1^ were considered as signatures for β-sheet structures, while bands centered between 1684 and 1695 cm^−1^ were assigned to β-turn structures. As seen, the spectral profiles of *Cnm*^(+)^*Sm* and *Cnm*^(−)^*Sm* strains in the Amide I region appear at a glance very different. A quantitative analysis of the area subtended by each Raman band revealed that *α*-helix was the dominant secondary structure in the protein composition of the *Cnm*^(−)^*Sm* strain (I_1660_/I_1649_ ≅ 0.1; cf. also fractional data in inset to Figure 6), with only a weaker random coil component being detected. On the other hand, in the protein structure of the *Cnm*^(+)^*Sm* strain, the ratio between random coil and α-helix configurations was I_1660_/I_1649_ ≅ 1.9 (i.e., close to the I_1264_/I_1297_ ≅ 1.7 ratio measured in the Amide III region; cf. above). In other words, comparisons of both Amide I and Amide III data between *Cnm*^(+)^*Sm* and *Cnm*^(−)^*Sm* strains consistently pointed to a high degree of disorder in the *Cnm* protein structure. In a recent paper by di Cologna et al. (2021), the presence was predicted of unstructured acidic and glycosylated threonine-rich *Cnm* domains, which protrude outward the β-sheet collagen-binding domain. Those authors proposed that the disordered threonine-rich repeat was necessary for maintaining structural stability in the *Cnm*^(+)^*Sm* bacterial cells, especially in acidic environments. Such an overall view of the *Cnm* structure seems to agree with our present Raman findings in the Amide I, II, and III regions, which unfolded the presence of both an acidic environment and a higher degree of protein disorder in the *Cnm*^(+)^*Sm* strain as compared to the *Cnm*^(−)^*Sm* one.

Finally, in examining the spectral features present in the *Cnm*^(−)^*Sm* strain but missing in the *Cnm*^(+)^*Sm* one, only one relative weak band was located at around 1512 cm^−1^ (cf. labels in Figure 6a). This band could be assigned to D-glucosamine (She et al., 1974) and its presence is in line with the findings discussed in Zones I and II.

### 3.4 Raman analyses of biofilm structures

The formation of bacterial biofilms involves the production of organic extracellular polymeric substances capable of linking with cells and adhering to soft and hard tissues. Biofilms, which are mainly comprised of polysaccharides, proteins, extracellular nucleic acids, lipids, and other biomolecules peculiar to individual strains, provide a three-dimensionally protected enclave supporting bacterial growth (Flemming and Wingender, 2010). Generally rich in negatively charged moieties and hydrophobic groups, a biofilm matrix also contains pores that facilitate the transport of nutrients (van Acker et al., 2014). In a recent paper (Pezzotti et al., 2023b), we have examined the *in vitro* formation of biofilm in *S. mutans* culture by means of Raman spectroscopy assisted by a deconvolution-based machine-learning algorithms. The insights gained in that study shall serve as a guide in the present analysis.

#### 3.4.1 Zone I—low wavenumber region (200~700 cm^−1^)

**Figure 7:**
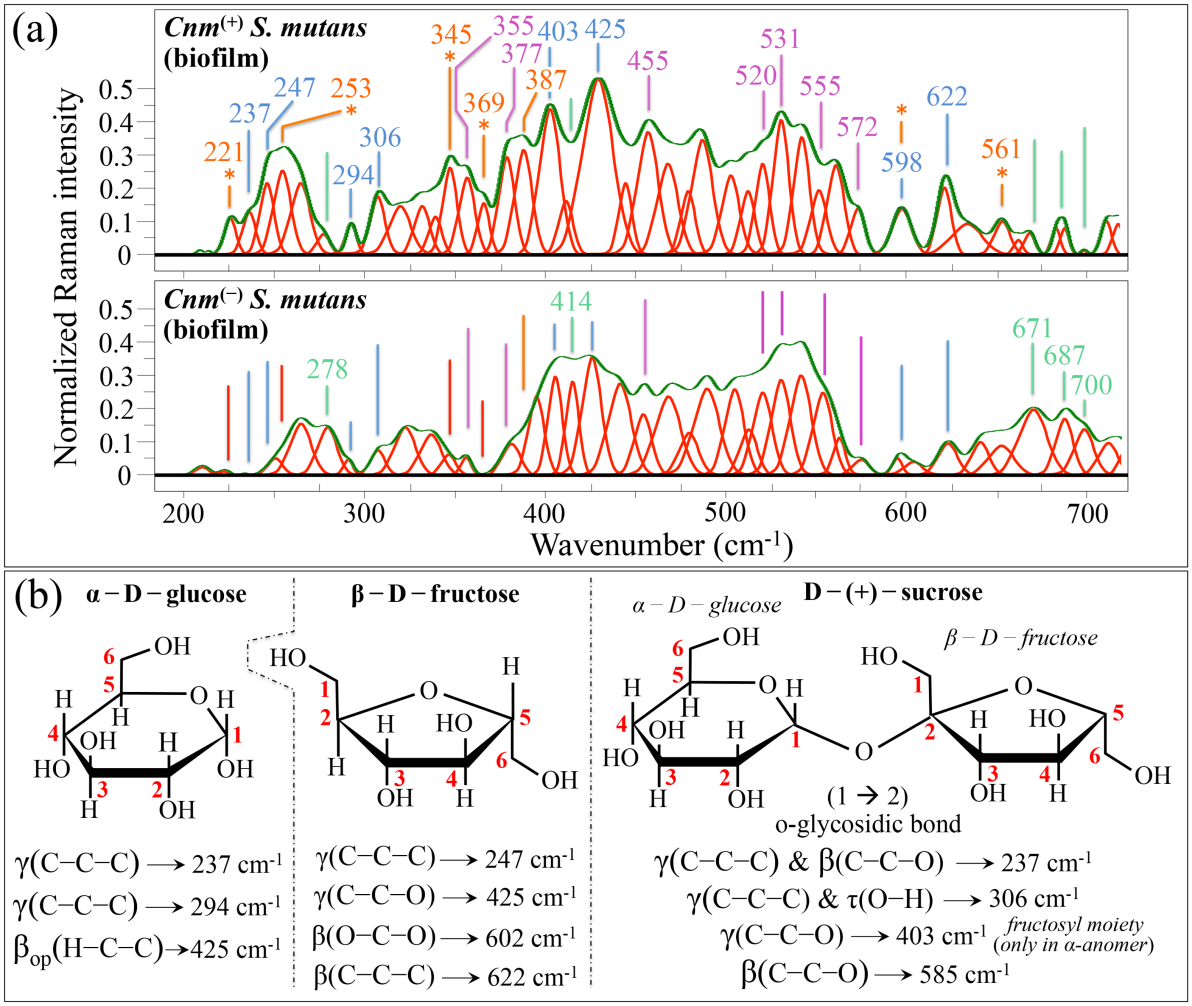
**(a)** Average Raman spectra of *Cnm*^(+)^*Sm* (upper) and *Cnm*^(−)^*Sm* (lower) cells cultured with sucrose in the wavenumber interval 200–700 cm^−1^ (Zone I). Both spectra, which mainly belong to biofilm structures, are deconvoluted into Lorentzian-Gaussian bands (cf. band wavenumbers in inset). The main molecular features discussed in the text are emphasized in inset to each spectrum. In **(b)**, vibrational origins are shown for signals belonging to α-D-glucose, β-D-fructose, and D − (+)— sucrose (symbols are the same as those shown in Figures 4, 5). Wavenumbers are in cm^−1^.

Looking at the difference diagram in Figure 3, one might notice a preponderance of Raman signals in the biofilm of the *Cnm*^(+)^*Sm* strain as compared to that of the *Cnm*^(−)^*Sm* one for the wavenumber interval 200~620 cm^−1^ (in purple color) and vice versa in the interval 620~700 cm^−1^ (in orange color). The wavenumbers of signals only present (or significantly preponderant) in the spectrum of the *Cnm*^(+)^*Sm* strain are listed in inset to Figure 7a. In the wavenumber interval 200 ~ 620 cm^−1^, one can yet find four bands (at 221, 253, 345, 369, and 387 cm^−1^), which are mainly contributed by sulfur compounds, as already discussed for isolated cells in the contest of Figure 2. Additional 13 bands are either peculiar or strongly enhanced in the spectrum of *Cnm*^(+)^*Sm* strain (cf. labels in inset to Figure 7a). To explain the origin of these bands, one needs to scrutinize the components of the extracellular polysaccharides of *S. mutans*. These are reported to typically include two types of glucans, namely, *α*-1,3- and α-1,6-glucan synthesized by glucosyltransferases, and fructans, which result from fructosyltransferase (Lemos et al., 2013). Glucans support adhesion of bacteria to tissues (Schilling and Bowen, 1992), while fructans serve as short-term energy storage (Burne et al., 1996).

Looking for fingerprint signals of specific polysaccharide components, the signals located at 355 cm^−1^ (C1–C2–C3 deformation) and 455 cm^−1^ (C5–C6–OH deformation) can only be found in the α-1,3-glucan structure, the 377 and 555 cm^−1^ (C3–C4–C5 deformation and C2–C1–O bending, respectively) bands belong to both α-1,3- and α-1,6-glucan structures, while the band located at ~520 cm^−1^ (C2–C3–OH bending) should only appear in the α-1,6-glucan structure (for the structure of glucans, cf. next subsection). Two additional bands found at 531 and 572 cm^−1^ correspond to C2–C3–O(–C1) and C5–C6–O(–H) bond deformation, respectively, and are both expected in *α*-1,3-glucans (Pezzotti et al., 2023b). Their enhancement likely arises from the formation of shortened oligomers, to the presence of glucofuran rings, and/or to C–C–C bending or C–O torsion in glycogen (*α*-1,4 glycosidic bonding) (Wiercigroch et al., 2017). In the spectrum of the *Cnm*^(−)^*Sm* strain, Raman signals in the interval 510 ~ 580 cm^−1^ might also be contributed by skeletal modes in *GlcNAc* molecules (She et al., 1974). This point will be further discussed in the description of the following spectral Zone II.

Upon comparing the low-wavenumber Raman spectra of the three different mono- and disaccharide sugar molecules: glucose, fructose, and sucrose (cf. structures in Figure 7b) (Fernandez Pierna et al., 2011; Wiercigroch et al., 2017), it is possible to assign the signals at 247 and 622 cm^−1^ exclusively to fructose (C–C–C deformation and in-plane C–C–C bending, respectively), the signals at 306 and 403 cm^−1^ (C–C–C deformation/O–H torsion and endocyclic C–C–O deformation on the fructosyl moiety in the α-anomer, respectively) only to sucrose, and the signal at 294 cm^−1^ (C–C–C deformation) only to glucose. On the other hand, the signal at 237 cm^−1^ (C–C–C deformation and C–C–O bending) is common to both sucrose and glucose, but is missing in fructose, while the signal at and 425 cm^−1^ is common to glucose (H–C–C out-of-plane bending) and fructose (endocyclic C–C–O deformation on the fructosyl moiety in the β-anomer), but absent in sucrose. The relatively broad signal observed at 598 cm^−1^, which experiences a stronger intensity in the spectrum of the *Cnm*^(+)^*Sm* strain, could be considered as a composite band from two distinct contributions, namely, 585 (C–C–O bending) and 602 cm^−1^ (O–C–O bending) from sucrose and fructose, respectively (Wiercigroch et al., 2017). All the above-mentioned vibrational modes are summarized in Figure 7b.

Five signals located at 278, 414, 671, 687, and 700 cm^−1^, were peculiar to (or clearly enhanced) in the spectrum of the *Cnm*^(−)^*Sm* strain (cf. labels in inset to Figure 7a). All these signals were conspicuously missing in the spectrum of *Cnm*^(−)^*Sm* bacteria in absence of biofilm (cf. Figure 4a); therefore, they should necessarily belong to biofilm molecules. The concomitant enhancement of signals at 278, 414 and 700 cm^−1^ points to the presence of monosaccharide D-glucosamine and glycolipids in the biofilm of the *Cnm*^(−)^*Sm* strain (She et al., 1974; Krafft et al., 2005). On the other hand, the bands at 671 and 687 cm^−1^ (symmetric stretching of the 6-ring and 5-ring deformation, respectively) could be traced back to extracellular DNA (i.e., guanine, adenine, and thymine overlapping vibrations) (Madzharova et al., 2016).

In summary, Raman analyses in the low-wavenumber interval suggested that the biofilm of the *Cnm*^(+)^*Sm* strain contained slightly (but detectably) higher amounts of glucans, while containing significantly higher amounts of mono and disaccharide glucose, fructose, and sucrose as compared to the biofilm of the *Cnm*^(−)^*Sm* strain. Note that this latter is an opposite trend as compared to that found in the cultures of bacteria alone (cf. Figure 4). In that case, the *Cnm*^(−)^*Sm* strain produced and endocytotically stored significantly higher fractions of glycogen molecules. On the other hand, the *Cnm*^(−)^*Sm* strain appeared to release larger amounts of extracellular DNA, thus contributing to a higher structural integrity and stability of the biofilm. The above points will be further discussed in the context of signal at higher wavenumbers.

#### 3.4.2 Zone II—intermediate wavenumber region (700~1200 cm^−1^)

**Figure 8:**
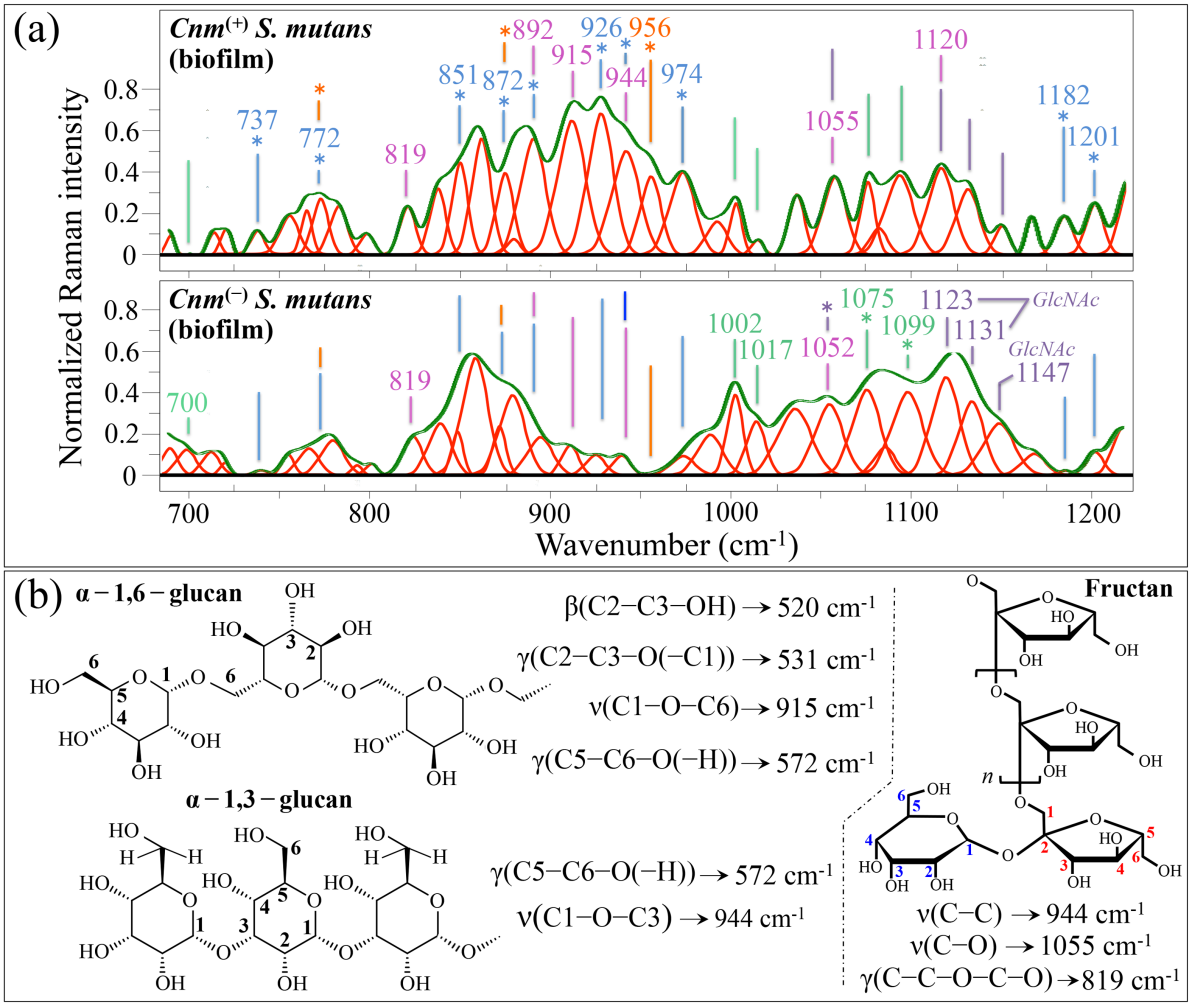
**(a)** Average Raman spectra of *Cnm*^(+)^*Sm* (upper) and *Cnm*^(−)^*Sm* (lower) cells cultured with sucrose in the wavenumber interval 700–1200 cm^−1^ (zone II). Both spectra, which mainly belong to biofilm structures, are deconvoluted into Lorentzian-Gaussian bands (cf. band wavenumbers in inset). The main molecular features discussed in the text are emphasized in inset to each spectrum. In **(b)**, vibrational origins are shown for signals belonging to α-1,6-glucan, α-1,3-glucan, and fructan structures (abbreviations and symbols are the same as those shown in Figures 4, 5). Wavenumbers are in cm^−1^.

In this wavenumber zone, the spectrum of the *Cnm*^(+)^*Sm* strain contained 14 bands not seen in or clearly enhanced as compared to the spectrum of the *Cnm*^(−)^*Sm* strain (cf. labels in inset to Figure 8a). The most prominent zone includes bands at 915 and 944 cm^−1^, which arise from C1–O–C6 stretching in *α*-1,6-glucans and C1–O–C3 stretching in α-1,3-glucans, respectively (cf. Figure 8b). The presence of these strong bands confirms the interpretation of the 520 cm^−1^ (C2–C3–OH bending) band from α-1,6-glucan structure and of the bands at 531 and 572 cm^−1^ (C2–C3–O(–C1) and C5–C6–O(–H) bond deformation) in the *α*-1,3-glucan structure, as discussed in the context of Zone I (Corbett et al., 1991; Synytsya et al., 2009). Note that the intensity ratio between bands at 915 and 944 cm^−1^, which represents the fractional amount of α-1,6-to α-1,3-glucans, does not appear to be significantly altered between the *Cnm*^(+)^*Sm* and *Cnm*^(−)^*Sm* strains (areal ratio ~0.77 for both strains). The signal at 892 cm^−1^ represents the correspondent C1–O–C3 stretching in β-1,3-glucan structure (Mikkelsen et al., 2010; Pezzotti et al., 2021c). However, this latter band can hardly be used as an exclusive fingerprint for the presence of the β-glucan structure because it also overlaps C–C signals from monosaccharides (Cael et al., 1974; Wiercigroch et al., 2017). According to Tahir et al. (2020), bacteria-produced exopolysaccharides include glucans and fructans in various fractions. These two classes of exopolysaccharides include both aldehydic and ketonic glycosidic monomeric linkages, such difference in linkage type leading to a variety of different conformations and related shifts in Raman spectral features (Orendorff et al., 2002). Among Raman bands only belonging to fructans in Zone II, prominent spectral features were found at 819 (C–C–O–C–O skeletal vibration), 944 (C–C stretching), 1055 (C–O stretching in fructofuranoid) and 1120 cm^−1^ (C–O stretching) (Balan et al., 2018). All these bands were indeed observed in both spectra of Figure 8a with similar relative intensities, except for the signal at 944 cm^−1^. This latter signal completely overlaps the C–C stretching signal from sucrose disaccharides, which was already shown to enrich the biofilm of the *Cnm*^(+)^*Sm* strain in the context of Zone I. In this same context, the bands at 737 (ring deformation in sucrose), 772 (C–C–C bending in glucose), 851 (CH_2_ torsion and C–C stretching in glucose α-anomer), 872 (CH_2_ torsion and C–C stretching in sucrose on glucose ring side), 892 (CH_2_ torsion and C–C stretching in glucose β-anomer), and 974 cm^−1^ (C-C-H and C-C-O bending in fructose) confirmed a predominance of monosaccharide and disaccharide signals in the *Cnm*^(+)^*Sm* strain as compared to the *Cnm*^(−)^*Sm* one (Wiercigroch et al., 2017). Regarding the band at 956 cm^−1^, which was completely missing in the spectrum of *Cnm*^(−)^*Sm* strain, it could be assigned to the signal from the SO_3_^2−^ stretching mode in sulfite ions present in the *Cnm* protein of bacterial cells (cf. Section 3.3) (Sato et al., 1985). This hypothesis is supported by the presence of a series of low-wavenumber bands in the interval 220~390 cm^−1^ also related to sulfur compounds peculiar to the *Cnm*^(+)^*Sm* strain (cf. Figure 7a).

The wavenumber region between 1000 and 1150 cm^−1^ included stronger signals in the spectrum of *Cnm*^(−)^*Sm* strain as compared to the *Cnm*^(+)^*Sm* one (cf. Figures 3, 8a). Main contributions from signals at 1002 and 1017 cm^−1^, assigned to ring vibrations in phenylalanine, suggest a higher amount of cells in the biofilm of the *Cnm*^(−)^*Sm* strain, an observation in agreement with the finding, reported above in the analysis of Zone I, of a higher level of released extracellular DNA (Kim et al., 2018). Although this appears to be the most obvious explanation for the reduced signals of phenylalanine, the possibility that these signals be contributed by different compounds containing the benzene ring and peculiarly related to *Cnm*^(+)^*Sm* strain metabolism will be discussed in the forthcoming Section 4.1. Other prominent bands for the *Cnm*^(−)^*Sm* strain in this region were found at 1075, 1099, 1123, 1131, and 1150 cm^−1^. The two bands at 1075 and 1099 cm^−1^ can be associated to O–P–O stretching vibrations on DNA backbone, which further strengthens the hypothesis of higher levels of extracellular DNA in the biofilm of *Cnm*^(−)^*Sm* bacteria. The three remaining signals at 1123, 1131, and 1150 cm^−1^ point to a common origin from *GlcNAc* molecules (She et al., 1974), as already hinted in the previous description of Zone I.

In summary, signals from the spectroscopic Zone II confirm the finding reported above for Zone I, as follows: (i) the biofilm fabricated by the *Cnm*^(+)^*Sm* strain contained higher amounts of glucans; however, the ratio between α-1,6- and α-1,3-glucan fractions (namely, the intensity ratio between bands at 915 and 944 cm^−1^) did not show any significant variation as compared to that recorded for the *Cnm*^(−)^*Sm* strain; (ii) the *Cnm*^(+)^*Sm* strain’s biofilm contained clearly higher amounts of mono and disaccharide glucose, fructose, and sucrose as compared to the *Cnm*^(−)^*Sm* strain’s one; (iii) the presence of signals from sulfur compounds was confirmed also in Zone II; (iv) the *Cnm*^(−)^*Sm* strain appeared to contain a higher amount of cells and extracellular DNA in its biofilm as compared to *Cnm*^(−)^*Sm* strain; and (v) higher amounts of *GlcNAc* stemmed in the biofilm of the *Cnm*^(−)^*Sm* strain, as recorded in the spectral interval 1100 ~ 1150 cm^−1^.

#### 3.4.3 Zone III—high wavenumber region (1200~1700 cm^−1^)

**Figure 9:**
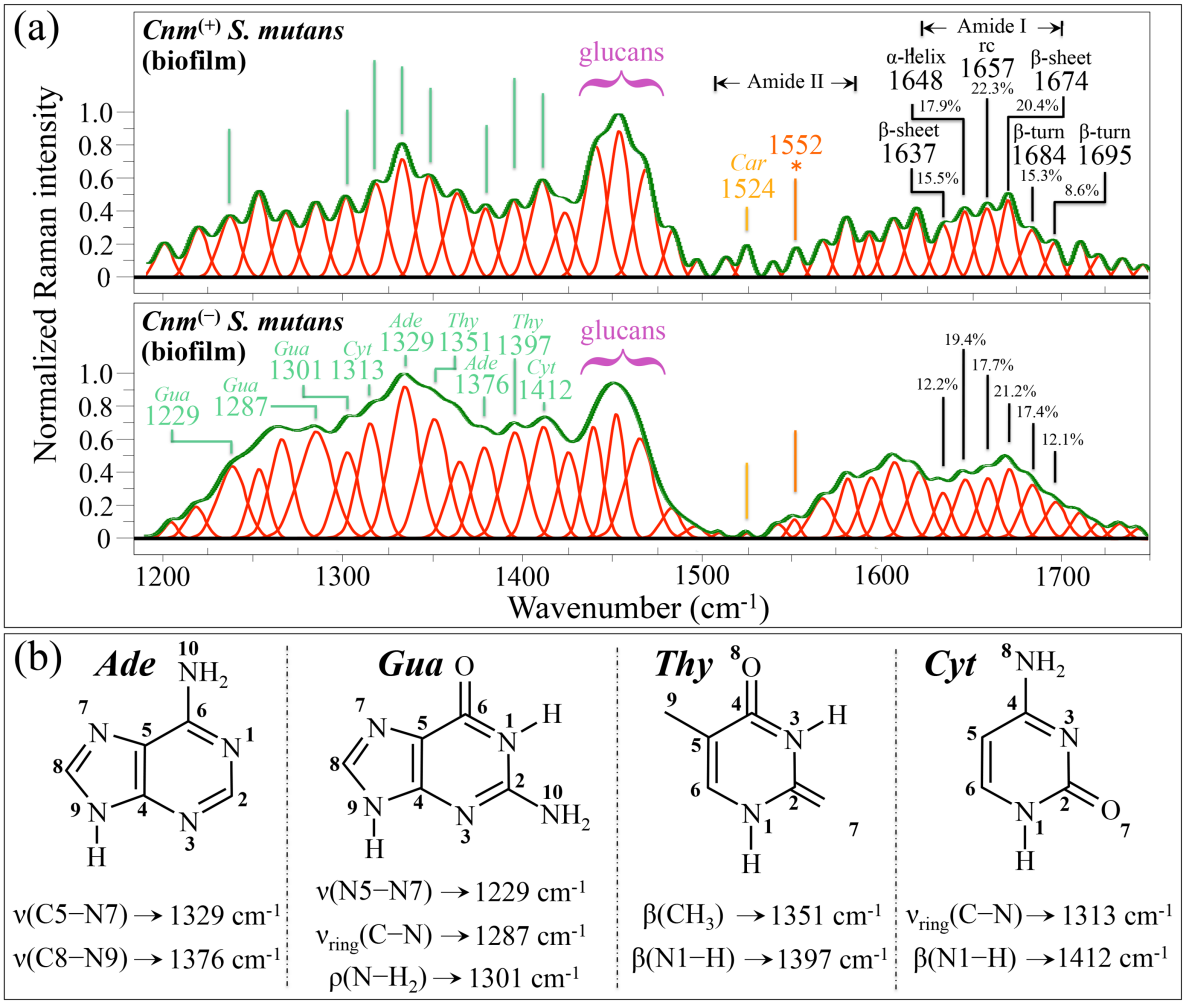
**(a)** Average Raman spectra of *Cnm*^(+)^*Sm* (upper) and *Cnm*^(−)^*Sm* (lower) cells cultured with sucrose in the wavenumber interval 1200–1800 cm^−1^ (zone III). Both spectra, which mainly belong to biofilm structures, are deconvoluted into Lorentzian-Gaussian bands (cf. band wavenumbers in inset). The main molecular features discussed in the text are emphasized in inset to each spectrum. In **(b)**, vibrational origins are shown for signals belonging to adenine (*Ade*), guanine (*Gua*), thymine (*Thy*), and cytosine (*Cyt*) (*Car*➔ carotenoid; symbols: ν_ring_ ➔ stretching within ring; the other abbreviations and symbols are the same as those shown in Figures 4, 5). Wavenumbers are in cm^−1^.

Upon screening the difference plot for biofilm spectra in Figure 3, it appears immediately clear that the spectral zone 1230~1425 cm^−1^ is clearly more prominent in the spectrum of the *Cnm*^(−)^*Sm* strain. Immediately ahead of this zone and below 1560 cm^−1^, the opposite trend is observed with signals in the spectrum of the *Cnm*^(+)^*Sm* strain becoming prominent. As already discussed in the Zone III of spectra from cells (Figure 6a), the spectral zone between 1230 and 1425 cm^−1^ incorporates a number of overlapping signals from Amide III and amino acid residues belonging to bacterial proteins (Schweitzer-Stenner et al., 2002; Zhu et al., 2011). However, an explanation based on these assignments, although sound for bacterial cells cannot be applied to biofilm spectra, because the total amount of proteins, as estimated from the signal intensity of the adjacent Amide I zone between 1637 and 1695 cm^−1^ (mainly related to protein vibrations in the amide plane) is ~6% higher in the spectrum of the *Cnm*^(+)^*Sm* strain (for slightly modified fractions of secondary structures; cf. Figure 9a). A possible explanation for this discrepancy could be given by considering that the spectral region 1230~1425 cm^−1^ includes some of the most intense signals from purine and pyrimidine molecules in extracellular DNA (Madzharova et al., 2016), whose higher fraction in the *Cnm*^(−)^*Sm* biofilm sample was already pointed out in the above description of the biofilm Zones I and II (cf. Figure 9b for band assignments).

An apparently minor spectral detail, but yet an important one in the biofilm context, was found in the Amide II region: a signal at 1524 cm^−1^ could clearly be detected only in the spectrum of the *Cnm*^(+)^*Sm* strain (cf. Figure 9a). The C=C stretching vibration at 1524 cm^−1^ is the main sensitive marker for a number of different carotenoid molecules (Udensi et al., 2022). Other main carotenoid signals are expected at around 1158 and 1004 cm^−1^, which, however are in strong overlap with signals from other molecules and thus cannot be equally assumed as markers for carotenoids. *Streptococci* are known for their capacity of synthesizing carotenoids in biofilm, the higher the amount of glucose the higher the content of synthesized carotenoids (Taylor and Davies, 1976). This seems to indeed be the case of the *Cnm*^(+)^*Sm* strain (cf. intensity of the glucose-related bands in Zone I). Carotenogenesis is enhanced by reactive oxygen species (ROS) generated under stress conditions (Taylor and Davies, 1976). Unlike the case of aerobes, in which biofilm aeration leads to the production of normal carotenoids, an increased oxygen tension in cultures of facultative anaerobes (such as *S. mutans*) rather results in carotenoid degradation. This latter observation points to a stronger and more protective biofilm structure in the *Cnm*^(+)^*Sm* strain, as also expected by a higher amount of glucans (cf. Figure 3). Finally, the band seen at ~1552 cm^−1^, which, as previously discussed, could be tracked back to indoxyl sulfate, appears stronger in the spectrum of the *Cnm*^(+)^*Sm* biofilm and the only available fingerprint for this molecule in *S. mutans* biofilm samples.

## 4 Discussion

### 4.1 Updating structure and binding characteristics of *Cnm* protein

According to the present knowledge on *Cnm*^(+)^*Sm* strains (Nomura et al., 2013; Esberg et al., 2017; di Cologna et al., 2021; Naka et al., 2022), *Cnm* is a cell surface protein composed of several domains, including an N-terminal signal sequence that targets the protein for secretion, the so-called *A* domain, which is crucial for binding to collagen, a proline-rich region and other domains that facilitate attachment to the bacterial cell wall, and sortase recognition sequences that anchor the protein to the cell wall peptidoglycan. The *A* domain of the *Cnm* protein specifically recognizes and binds to the triple-helical structure of collagen. This domain contains binding motifs that interact with the repeating glycine-X-Y sequences of collagen, where X and Y are often proline and hydroxyproline. The present Raman results confirmed this view of the *Cnm* protein since prominent signals from glycine and proline residues were clearly detected in the Raman spectrum of *Cnm*^(+)^*Sm* cells (cf. Figures 5a, 6a). Collagen binding has so far been considered to involve a combination of hydrogen bonds (between amino acid side chains in *Cnm* and triple helix), electrostatic interactions (between oppositely charged residues in *Cnm* and collagen), and possibly hydrophobic interactions. These latter interactions further stabilize the bonds between *Cnm* protein and collagen. Regarding binding specificity, the *Cnm* protein preferentially binds to the triple-helix of collagen, which is the predominant collagen form in connective tissues. This is the reason why *S. mutans* can strongly adhere to collagen-rich tissues such as blood vessels and heart valves, such bond facilitating the invasion of endothelial cells, leading to potential vascular damage, and contributing to diseases such as endocarditis and cerebral microbleeds (Naka et al., 2022).

Despite the above significant body of structural information, however, there has so far been no study reporting that the *Cnm* protein of *S. mutans* is rich in oxysulfur molecules, and suggesting that it might also use sulfur moieties to bind to collagen. The unique sensitivity of Raman spectroscopy in detecting sulfur-related bonds enabled to inconfutably prove the presence of this new adhesion path.

The notion of sulfide microbial oxidation, as arising from a complex network of pathways leading to the synthesis of intermediate sulfur species (and partly back to sulfate), is well known in microbiology (Dahl, 2000; Friedrich et al., 2001). Sulfur intermediates include elemental sulfur, polysulfides, thiosulfate, and sulfite, which in turn trigger further microbial oxidation, and reduction or disproportionation reactions. A common mechanism was proposed (Friedrich et al., 2001), according to which an oxidized sulfur anion binds covalently to cysteine residues in a specific protein to form S-thiocysteine. The outer sulfur atom is then oxidized by sulfur dehydrogenase and sulfate hydrolyzed by sulfatase. Genome sequence data for sulfur-oxidizing bacteria pointed to the presence of similar proteins in both anaerobic phototrophic and aerobic lithotrophic bacteria, implying that oxidation of sulfur to sulfate may actually be mediated by very similar pathways in quite different bacteria, with differences merely related to specificities of sulfur substrates and to the mechanism of linkage of the sulfur atom to be oxidized. The present Raman study suggests that *Cnm*^(+)^*Sm* cells might follow a similar pathway, with the *Cnm* protein being part of the protein family with sulfur-oxidizing ability.

Cysteine and methionine cover crucial roles in bacterial cell metabolisms because of the high reactivity of their sulfur group, which plays an essential role in the catalytic sites of many enzymes and participates in ion and redox metabolisms (Ayala-Castro et al., 2008). Regulation of S-related pathways involves a large variety of mechanisms in different groups of bacteria evolving faster than many other regulatory pathways. Sperandio et al. (2010) reported that the *S. mutans* genome encodes four LysR-type transcriptional regulators, three of which, MetR, CysR (cysteine synthesis regulator), and HomR (homocysteine synthesis regulator), are phylogenetically related. The latter two regulators included two conserved motifs suggesting their role in the regulator binding recognition site, according to a global sulfur amino acid supply gene regulatory pathway. Such a pathway is key in adaptation of *S. mutans* to new environments, which might require acquisition of new functions (enhanced adhesiveness in the present specific case). A rapid drift of regulatory function in the *Streptococcaceae*, as we observed in this study, should be considered as an additional paradigm of evolution, enabling adaptation to different niches within hosts and shaping their pathogenic behavior. In summary, the results of the present Raman study support the interpretation of fast and efficient evolution in *S. mutans* bacteria in order to better exploit adhesiveness. Future genomic studies will serve to validate the present findings.

### 4.2 Similarities and diversities with uremic toxins

Accumulation in the bloodstream of retention-protein solutes is known to be the origin of a number of different pathologies (Rozen et al., 2001; Opdebeeck et al., 2020; Nemet et al., 2023). Besides differences in molecular structures related to specific pathologies, these toxins share difficult removal characteristics as a consequence of both their high molecular weight and strong bonding characteristics with soft tissues. In studies on kidney diseases by Evenepoel et al. (2009) and Vanholder et al. (2003), indoxyl and *p*-cresyl sulfates were located as key protein-bound toxins originating from protein fermentation in the intestine. In kidney diseases, the intestinal microbiota first promotes the breakdown of tyrosine/phenylalanine and tryptophan to successively form *p*-cresol and indole, respectively, upon oxidation and conjugation with sulfate. In a self-sustaining degenerative loop, indoxyl and *p*-cresyl sulfates increasingly accumulate in the bloodstream, bind to albumin and soft tissues, further deteriorate kidney functionality, and become the cause of cardiovascular diseases and mortality (Evenepoel et al., 2009; Lin et al., 2015; Kaminski et al., 2017). It should be noted that, in the case of the above-described pathological pathway, indoxyl and *p*-cresyl sulfate molecules are both generated starting from byproducts of bacterial fermentation of aromatic amino acids. In the case of indoxyl sulfate, tryptophan is degraded into indole by microbial tryptophanase genes encoded in the genome of a variety of gut commensals (Cozzolino et al., 2018), while *p*-cresyl sulfate originates from metabolization of protein-derived tyrosine by gut microbes (Devlin et al., 2016). In both cases, enzymatic absorption in the host followed by sulfation results in the final compound (Brix et al., 1999). Therefore, the synthesis of both indoxyl and *p*-cresyl sulfates is a process made it possible by a community of distinct but interacting biological entities, namely, a “metaorganism” including gut microbiota and host-dependent enzymatic processes. In a recent paper, Nemet et al. (2023) thoroughly identified the pro-thrombotic gut microbiota and microbial enzymes involved in the generation of indoxyl and *p*-cresol sulfates. Those researchers also showed which gut microbial genes suffice in the generation of such uremic toxins, and coupled experimental findings with large-scale clinical observations in successfully linking blood levels of uremic toxins to 5-year mortality predictions. This appears to be the last update of a series of detailed studies attempting to unfold the complexity of pro-thrombotic gut microbiota-generated metabolites and pathways (Dodd et al., 2017; Cozzolino et al., 2018; Robert et al., 2018; Skye et al., 2018; Gupta et al., 2020; Organ et al., 2020; Zhu et al., 2021; Buffa et al., 2022; Needham et al., 2022; Stewart Campbell et al., 2022; Zhu et al., 2023; Nemet et al., 2020; Wilborn et al., 1993).

**Figure 10:**
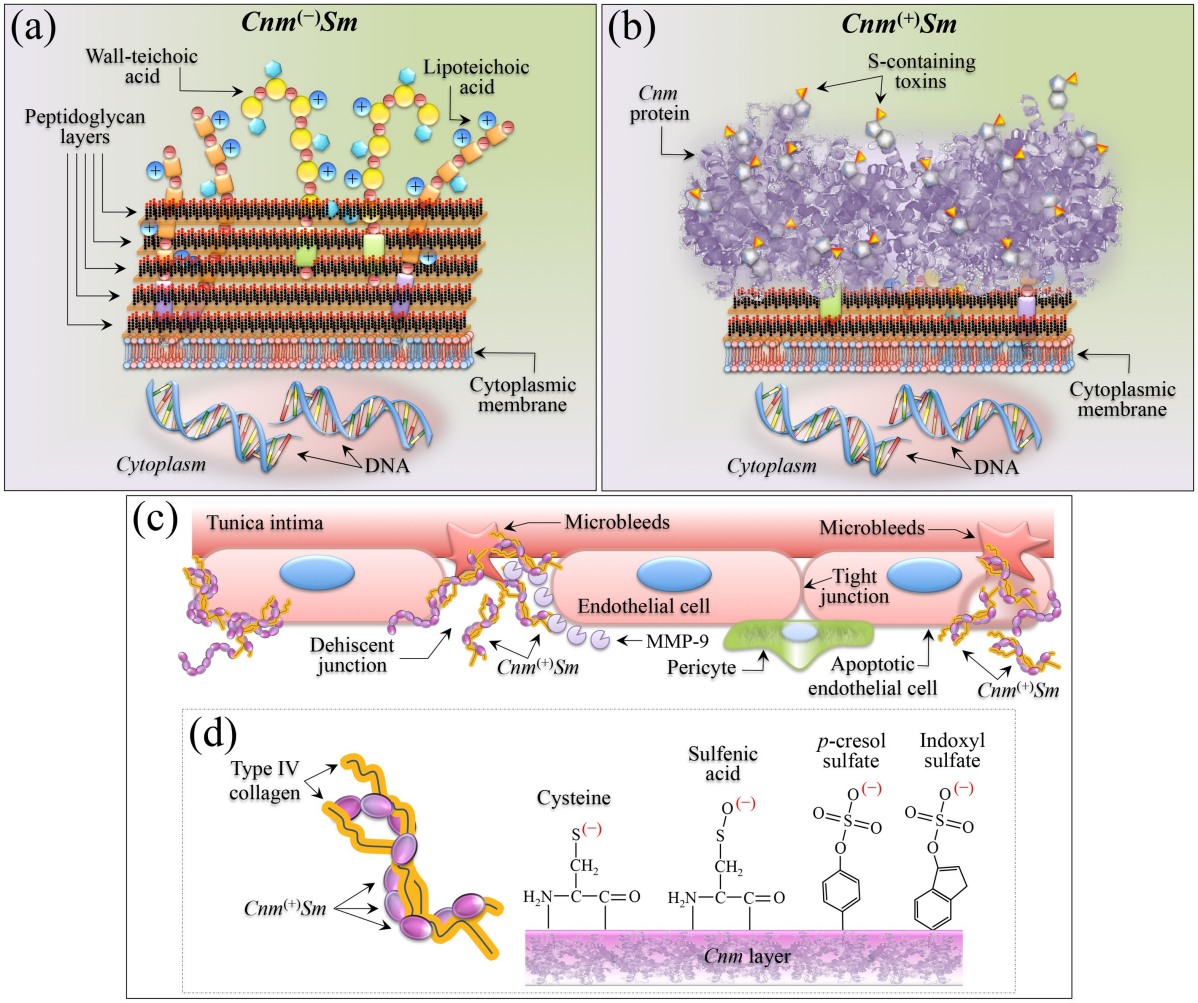
Drafts summarizing the main structural differences between *Cnm*^(−)^*Sm* **(a)** and *Cnm*^(+)^*Sm* **(b)** bacterial cells; **(a)** shows a structure rich in peptidoglycans, wall-teichoic and lipoteichoic acids (both composed of a linkage, repeat, phosphate, D-alanylation, and glycosylation units), while **(b)** launch a highly disordered *Cnm* protein layer wrapping the cell and a significant simplification of the peptidoglycan wall-structure (including teichoic and lipoteichoic moieties). The highly disordered *Cnm* protein in **(b)** is replenished of toxins exhibiting sulfenic and sulfonic acid termini. In **(c)**, a draft is offered of the mechanism by which blood-circulating *Cnm*^(+)^*Sm* bacteria causes intracerebral hemorrhage: binding to denuded basement membranes at dehiscent cellular junctions (left side) and direct invasion of endothelial cells (right side). Collagen-adhesion characteristics through sulfur chemistry are depicted in **(d)**: direct attachment to type IV collagen through S–S bonding by cysteine and sulfenic acid to indirect pathways including hyperglycemia-inflammation-coagulation induced by indoxyl sulfate and *p*-cresol sulfate moieties.

In the stream of many clinical studies of *S. mutans* strains (Miyatani et al., 2015; Shiga et al., 2021; Benson et al., 2023; Ikeda et al., 2023), the results of the present Raman experiments revealed new insight into the molecular mechanism behind the pro-thrombotic path caused by oral bacteria. A striking similarity with the path of gut microbiota-related uremic diseases consisted in the fact that also *Cnm*^(+)^*Sm* bacteria appear to exploit sulfur compounds (i.e., indoxyl and *p*-cresol sulfates, and other oxysulfur anions) for enhancing molecular adhesiveness. However, a fundamental difference with the strategy of gut bacteria consisted in the fact that the *Cnm*^(+)^*Sm* strain did not need to borrow enzymatic reactions from the host in order to create sulfur toxins in the *Cnm* protein structure and related thrombus generation. Figure 10 summarizes the main structural differences, as revealed by Raman spectroscopic analysis, between *Cnm*^(+)^*Sm* and *Cnm*^(−)^*Sm* bacteria, and suggests a possible (direct) mechanism of bacterial adhesion to soft tissue. As seen in comparing Figures 10a,b, the formation of a highly disordered *Cnm* protein layer in the *Cnm*^(+)^*Sm* strain occurs together with a significant simplification of the peptidoglycan wall-structure (including reduced amounts of teichoic and lipoteichoic moieties) as compared to the *Cnm*^(−)^*Sm* strain. The *Cnm* protein is replenished of toxins exhibiting sulfenic and sulfonic acid termini (cf. Figure 10b). In any protein containing a deprotonated cysteine residue, oxidation by oxygen radicals (i.e., H_2_O_2_) could induce reversible/covalent modifications of cysteine thiolate residues located in active and allosteric sites, the sensitivity to oxidation thus depending on the ionization constant (pKa) and the local environment of the cysteine residue (Breton-Romero and Lamas, 2014; Hosoki et al., 2022). The majority of cysteine residues display a high value of ionization constant for the sulfhydryl group (pKa = 8.5), thus being hardly deprotonated and remaining inert to H_2_O_2_ oxidation. However, there are exceptions to this general trend, with specific proteins exhibiting a pKa value as low as 5.6. Such proteins are quite reactive under pH physiological conditions with cysteine thiolate anions being prone to first react with H_2_O_2_ to form unstable intermediate cysteine sulfenic acid (R–S–OH), and to successively produce disulfides (R–S–S–R) or sulfonic acid moieties (considered to be biologically irreversible) (Claiborne et al., 1993; Veal et al., 2007). The present Raman experiments prove that the *Cnm*^(+)^*Sm* bacteria are capable to produce such highly reactive protein residues in their *Cnm* structure. More specifically, the analysis of the *Cnm*^(+)^*Sm* spectrum in Section 3.3 uncover both the presence of disulfide bonds across cysteine residues of the same protein (i.e., intramolecular disulfide bonds; cf. the strong band at 253 cm^−1^ in Figure 4a) (Denu and Tanner, 1998) and the formation of oxysulfur terminals with potential to link with cysteines located in different molecules to produce homo- or hetero-dimers by intermolecular disulfide bond (Lee et al., 2002). Note that disulfides can also give rise to a mixed-disulfide between glutathione and the thiol of another protein (a reaction usually referred to as S-glutathionylation). The presence of low-wavenumber signals peculiar to glutathione only in the spectrum of *Cnm*^(+)^*Sm* (cf. Figures 4a, 6a) indeed hints to the S-glutathionylation pathway. Moreover, reaction with amides is also possible to form sulfenyl amide (–SN–) (van der Wijk et al., 2004). In this latter context, a block in glycolysis was reported for *S. mutans* (and other oral bacteria) upon lactoperoxidase-thiocyanate-hydrogen peroxide reaction (Finkel, 2012). Inhibition of glucose transport occurs as a consequence of oxidation of sulfhydryl groups to yield sulfenic acid and sulfenyl thiocyanate derivatives (Mickelson, 1979; Carlsson et al., 1983). Given the possible implications of this circumstance in treating *Cnm*^(+)^*Sm* pathologies, further studies should be conducted to elucidate whether glucose transport inhibition could also be possible for *Cnm*^(+)^*Sm* strains.

The collagen IV family, which has been reported as the main binding moiety in *Cnm*^(+)^*Sm* bacteria (Thomas and Aune, 1978; Abranches et al., 2011; Nakano et al., 2011), comprises six highly homologous *α*-chains, each containing an amino-terminal domain rich in cysteine and lysine residues. The presence of cysteine- and lysine-rich residues at the amino terminus is essential for interchain crosslinking of four triple-helical molecules through disulfide bonds and lysine-hydroxylysine crosslinks (Khoshnoodi et al., 2008). The present Raman data suggests that *Cnm* cysteine residues are prone to form disulfide bonds, a circumstance that could also occur in binding interactions between the *Cnm* protein and collagen IV. The proposed mechanism by which blood-circulating *Cnm*-expressing *S. mutans* causes intracerebral hemorrhage includes two possible paths: (i) binding to denuded basement membranes (mainly composed of collagen IV) upon exploiting damaged tight junctions (Thomas and Aune, 1978); and/or (ii) direct invasion of endothelial cells (Abranches et al., 2009; Nakano et al., 2011; Avilés-Reyes et al., 2014b; Nomura et al., 2020). In the context of (i), as depicted on the left side of Figure 10c, *Cnm*^(+)^*Sm* bacteria adhere to the denuded basement membrane via endothelial junction dehiscence, with subsequent migration of circulating neutrophils to the lesion. In the case (ii), *Cnm*^(+)^*Sm* bacteria adhere first to and then invade endothelial cells (cf. right side of Figure 10c). In both cases of endothelial junction-dehiscence and direct invasion paths, *Cnm*-binding to circulating type IV collagen was suggested to be instrumental to the induction of bacteremia, not only because it enhances bacterial adhesion probability to host endothelial surface, but also for its function in the successive host cell internalization and pro-inflammatory response (Shiga et al., 2021). The new insight of the present Raman study is represented by the finding that *S. mutans* enhances its collagen-adhesion characteristics through sulfur chemistry (cf. Figure 10d) in the context of an overall invasive strategy in the blood stream, independent of successive attachment loci in the host tissue. The presence of a full series of oxysulfur molecules at the bacterial surface involves a cascade of events spanning from direct attachment to type IV collagen and cells (Shiga et al., 2021) (through S–S bonding by sulfites) to indirect, but highly degenerative, pathways including hyperglycemia-inflammation-coagulation induced by indoxyl sulfate and *p*-cresol sulfate moieties (Rozen et al., 2001).

### 4.3 Raman insights into biofilm structures

In building biofilm structures, *S. mutans* synthesizes adhesive glucans from sucrose by means of glucosyltransferases (GTFs), which are enzymes capable to catalyze the formation of glycosidic bonds. Three main types of GTF in cooperative action are known: GTFB, GTFC, and GTFD (Ooshima et al., 2001). GTFB and GTFC are located at the cell surface and mainly synthesize water-insoluble glucans (i.e., rich in α-1,3-glucosidic linkages) (Aoki et al., 1986; Hanada and Kuramitsu, 1988), while GTFD synthesizes water-soluble glucans (i.e., rich in α-1,6-glucosidic linkages) and is also detected in culture supernatant (Hanada and Kuramitsu, 1989). Each GTF contains two distinct functional domains, namely, an amino-terminal catalytic domain, which binds/hydrolyzes the substrate of sucrose, and a carboxyl-terminal glucan-binding domain, which functions as an acceptor for binding glucan and decides the nature of the synthesized glucan (Mooser et al., 1988; Wong et al., 1990; Kato et al., 1992). Accordingly, simultaneous GTFB and GTFC glucan synthesis is key in establishing a functional biofilm matrix that, while high in density, also enhances both coherence with bacterial cells and adherence to external surfaces (Tamasada et al., 2004; Koo et al., 2010; Xiao and Koo, 2010).

The present Raman experiments unveiled that the biofilm fabricated by the *Cnm*^(+)^*Sm* strain contained an overall higher amount of glucans as compared to that built by *Cnm*^(−)^*Sm* strain under the same culture conditions (cf. Figure 8). However, the volumetric ratio between α-1,6- and α-1,3-glucan fractions (namely, the intensity ratio between bands at 915 and 944 cm^−1^) in *Cnm*^(+)^*Sm* and *Cnm*^(−)^*Sm* biofilms did not show significant variations. A common value ~0.77 was found for both strains, which corresponds to approximately 57.2% α-1,3- and 42.8% α-1,6-glucan fractions, according to previous calibrations (Pezzotti et al., 2023b). Although the ratio between *β*– and α-glucans is difficult to calculate (due to strong overlap of the 892 cm^−1^ β-glucan marker with signals from monosaccharides; cf. Figure 8), the present Raman results suggest that the terms of cooperative action among GTFB, GTFC, and GTFD in *Cnm*^(+)^*Sm* and *Cnm*^(−)^*Sm* strains are the same. In other words, an improvement of the biofilm structure in terms of impermeability and mechanical strength is not a priority in the bacterial evolution into *Cnm*^(+)^*Sm*. Note also that the higher amount of mono and disaccharides in the biofilm of the *Cnm*^(+)^*Sm* strain as compared to the *Cnm*^(−)^*Sm* strain’s one could similarly be related to the higher overall amount of glucans recorded in the former strain. According to Matsumoto-Nakano (2018), cell-associated GTF enzymes also mediate binding of *S. mutans* to *in situ* formed glucans and a variety of glucan-binding proteins. Such proteins contribute to development of optimal plaque biofilm, while binding to exopolysaccharides for construction and maintenance of a balanced environment.

Finally, the higher propensity of the *Cnm*^(+)^*Sm* strain to synthesize carotenoids in biofilm (cf. Figure 9), which is directly related to the higher amount of glucose (Taylor and Davies, 1976), is a marker for a biofilm environment rich in ROS generated under stress conditions. We speculate that such an environment might be linked to a programmed strategy of cell death, which is actually executed by ROS. The finding of a lower amount of cells (and extracellular DNA) inside of the *Cnm*^(+)^*Sm* biofilm (cf. Section 3.4) could be part of a strategy to keep “slim” the biofilm population and to invest nutritional contents in younger and more prosperous cells with better prospects of colonizing additional sites, while recycling nutrients from the biomass of dead cells.

## 5 Conclusion

We investigated by high spectrally resolved Raman spectroscopy the molecular structure of *S. mutans* expressing the *cnm* gene in comparison to the same bacterium lacking that gene. The *Cnm*^(+)^*Sm* strain, which enters the vasculature from the oral cavity, is capable to bind collagen in vascular walls and to create cerebral microbleeds progressing over time. A number of striking structural differences were found in comparing *Cnm*^(+)^*Sm* and *Cnm*^(−)^*Sm* strains, which gave important hints about the molecular origin of cerebral microbleeds. Perhaps, the most striking difference resided in the copious presence of residual oxysulfur molecules in the *Cnm*^(+)^*Sm* strain, likely delivered in the bumpy *Cnm* structure located on the cell surface. Several vibrational fingerprints unquestionably related to indoxyl sulfate and other sulfate molecules suggest that the *Cnm*^(+)^*Sm* strain exploits sulfur chemistry for anchoring purposes. On the other hand, the *Cnm*^(−)^*Sm* strain showed a superior ability in producing and storing glycogen molecules as compared to the *Cnm*^(+)^*Sm* one. This suggests a downregulation (or different regulation) of genes involved in glycogen metabolism in the *Cnm*^(+)^*Sm* strain, possibly because this strain allocates differently its resources in adaptive advantage for tissue invasive activity. This interpretation paired with a significant simplification of the peptidoglycan structure in *Cnm*^(+)^*Sm* strain: with *Cnm* proteins wrapping the bacterial cell, the protective function of the peptidoglycan layer becomes less critical and its production can be minimized. Regarding biofilm, the *Cnm*^(+)^*Sm* strain produced larger amounts of overall glucans despite storing a lesser number of cells; however, the ratio between α-1,6- and α-1,3-glucan fractions showed no variations in comparison to the *Cnm*^(−)^*Sm* strain, suggesting that reinforcing strength and impermeability of the biofilm is not a priority in *Cnm*^(+)^*Sm* functionality.

In linking microbial structure to human diseases, this study demonstrates that integrating clinical and mechanistic studies with *in situ* Raman spectroscopy gives a path forward toward personalized therapeutic interventions to reduce risks of cardiovascular or other metabolic diseases.

## Data Availability

The datasets presented in this study can be found in online repositories. The names of the repository/repositories and accession number(s) can be found in the article/Supplementary material.
