# Supplementary material for: Raman signatures of Cnm-positive Streptococcus mutans: I, the molecular origin of cerebral microbleeds
Source: Front Microbiol. 2026 Apr 27;17:1784125. doi: 10.3389/fmicb.2026.1784125 (PMC13160496; doi:10.3389/fmicb.2026.1784125)
Supplement: Supplementary file 1 [file Table_1.docx]

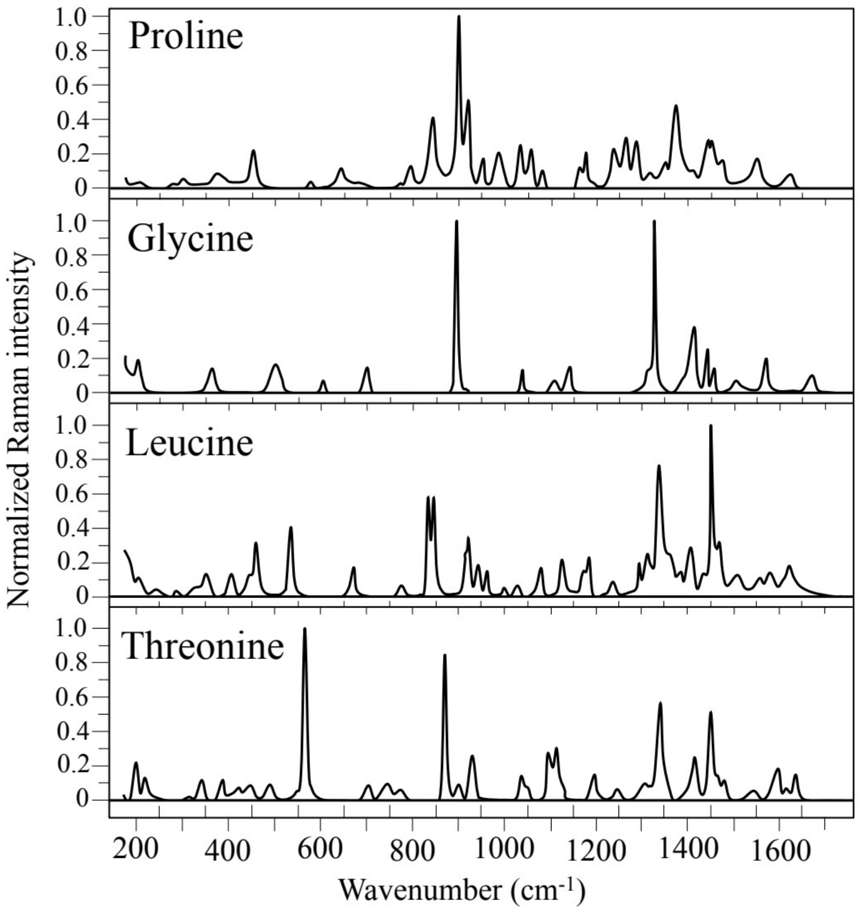

Proline
Glycine
Leucine
Threonine
Normalized Raman intensity
Wavenumber (cm-1)

Figure S1
